# *Neisseria meningitidis* Opc Invasin Binds to the Sulphated Tyrosines of Activated Vitronectin to Attach to and Invade Human Brain Endothelial Cells

**DOI:** 10.1371/journal.ppat.1000911

**Published:** 2010-05-20

**Authors:** Claudia Sa E Cunha, Natalie J. Griffiths, Mumtaz Virji

**Affiliations:** Department of Cellular and Molecular Medicine, School of Medical Sciences, University of Bristol, Bristol, United Kingdom; Institut Pasteur, France

## Abstract

The host vasculature is believed to constitute the principal route of dissemination of *Neisseria meningitidis* (Nm) throughout the body, resulting in septicaemia and meningitis in susceptible humans. *In vitro*, the Nm outer membrane protein Opc can enhance cellular entry and exit, utilising serum factors to anchor to endothelial integrins; but the mechanisms of binding to serum factors are poorly characterised. This study demonstrates that Nm Opc expressed in acapsulate as well as capsulate bacteria can increase human brain endothelial cell line (HBMEC) adhesion and entry by first binding to serum vitronectin and, to a lesser extent, fibronectin. This study also demonstrates that Opc binds preferentially to the activated form of human vitronectin, but not to native vitronectin unless the latter is treated to relax its closed conformation. The direct binding of vitronectin occurs at its Connecting Region (CR) requiring sulphated tyrosines Y_56_ and Y_59_. Accordingly, Opc/vitronectin interaction could be inhibited with a conformation-dependent monoclonal antibody 8E6 that targets the sulphotyrosines, and with synthetic sulphated (but not phosphorylated or unmodified) peptides spanning the vitronectin residues 43–68. Most importantly, the 26-mer sulphated peptide bearing the cell-binding domain _45_RGD_47_ was sufficient for efficient meningococcal invasion of HBMECs. To our knowledge, this is the first study describing the binding of a bacterial adhesin to sulphated tyrosines of the host receptor. Our data also show that a single region of Opc is likely to interact with the sulphated regions of both vitronectin and of heparin. As such, in the absence of heparin, Opc-expressing Nm interact directly at the CR but when precoated with heparin, they bind via heparin to the heparin-binding domain of the activated vitronectin, although with a lower affinity than at the CR. Such redundancy suggests the importance of Opc/vitronectin interaction in meningococcal pathogenesis and may enable the bacterium to harness the benefits of the physiological processes in which the host effector molecule participates.

## Author Summary

*Neisseria meningitidis* is a human pathogen that can cross the natural cellular barriers to reach the blood and the brain, causing septicaemia and meningitis. One of its surface molecules, Opc, has the capacity to attach to human cells lining the blood vessels. *In vitro*, the bacterium can do this by coating itself with the human serum factor vitronectin; and, by mimicking as vitronectin, it can bind to human cellular vitronectin-binding proteins (receptors). In this study, we have investigated the structural features of vitronectin that *N. meningitidis* recognises; such knowledge could help develop future strategies to control bacterial spread. We describe two different aspects of bacterial binding to vitronectin and demonstrate that one of these is a novel method, which occurs directly through the sulphated tyrosines of vitronectin available only when the molecule presents itself in an unfolded form. Such unfolded or activated vitronectin levels may be elevated during bacterial presence in the blood. Thus our observations imply that when bacteria appear in the blood, their presence may help to generate the form of vitronectin that they require for binding to and invading the cells that line human blood vessels to spread throughout body tissues including the brain.

## Introduction

Mucosal bacteria possess complex mechanisms of targeting their host cellular adhesion molecules and, in many cases, they adhere to specific host receptors or soluble effectors and consequently acquire enhanced colonisation and virulence potential. Understanding the molecular nature of such interactions may help identify possible targets of future strategies for prevention of infection or for altering the course of pathogenesis. In this study, we have analysed in detail, the molecular mechanisms by which *Neisseria meningitidis* (Nm, meningococcus), a human pathogen, interacts with serum vitronectin (Vn), which contains an RGD tripeptide sequence recognised by cellular integrins such as αvβ3 and αvβ5. As these may be expressed by endothelial cells throughout the body, this interaction has the potential to enable Nm to attach to various human vascular barriers, as has been demonstrated *in vitro* [1], [2].

Such Nm-endothelial interactions are particularly significant during the course of meningococcal pathogenesis; as, although Nm is a common mucosal coloniser (up to 30% of healthy human population may carry the organism), it is capable of causing disseminated infections in which the host vasculature provides the primary means of distribution to distal tissues. The process of breaching the vascular endothelial barrier may involve cellular entry, and invasion of endothelial cells of distinct origins as has been observed in a number of *in vitro* studies [2], [3], [4], [5]. It has been shown that meningococcal Opc protein, a major outer membrane adhesin, carries the property of cellular invasion, especially for endothelial cells [1], [2], [3], [4].

Meningococci are capsulate bacteria and the functional efficacy of their subcapsular integral outer membrane adhesins such as Opc (and Opa, a related but distinct class of opacity proteins) increases when bacteria become acapsulate or in situations when their cognate target cell surface receptor expression is enhanced. This occurs following conditions in the host that increase the circulation of inflammatory cytokines. Increased receptor density serves to overcome the inhibitory effects of bacterial surface polysaccharides [3], [6], [7], [8]. In addition, *in vitro* studies on unstimulated cells have also shown that both acapsulate and capsulate phenotypes of Nm may invade human cells in an Opc- or Opa- dependent manner [1], [2], [4], [6]. It appears that in the case of capsulate phenotypes, additional adhesins such as pili (fimbriae) that traverse the capsule greatly increase the efficiency of initial binding to target cells; this may then lead to secondary more efficient interactions via the subcapsular bacterial adhesins [4], [6], [9].

Opc-mediated interactions have also been shown to result in a level of bacterial transcytosis across the endothelial barriers *in vitro* [3], [10]. Although not universally present in Nm, Opc is expressed in numerous clinical isolates of Nm and retained by several meningococcal hypervirulent clonal lineages. Notably, it has been suggested that Opc expression may influence Nm pathogenic process thus altering the clinical profile of meningococcal disease. For example, Nm strains of the ET-37/ST11 clonal complex that lack the *opc* gene [11], [12] have been reported to cause serious cases of septicaemia [13], [14] but have a relatively low tendency to cause meningitis. This has led to the notion that Opc expression may enhance the bacterial ability to cause meningitis; perhaps suggesting an important role of the Opc protein in breaching the endothelial barrier to enter the brain [4]. This notion was further supported by the latter study that used Opc-deficient and Opc-expressing isolates of meningococcal clonal complexes ET-37/ST11 and ET-5/ST32 respectively [15]; the former were not able to cross human brain microvascular cell (HBMEC) monolayers *in vitro* [4]. Opc-deficient bacteria in our earlier studies also did not invade human umbilical vein endothelial cells (HUVECs) [2], [3].

Opc is a transmembrane protein of the beta barrel family with five surface-exposed loops. The protein is basic in nature and has a prominent surface loop 2. Together, the surface loops of Opc may form a positively charged crevice that may accommodate negatively-charged molecules [16], [17], [18]. More recent studies suggest that an induced fit mechanism, involving conformational changes in Opc, may operate for the recognition of molecular targets by the adhesin [19]. Opc has been shown to bind to heparin-like molecules and to heparan sulphate proteoglycans (HSPG) on human epithelial cells *in vitro* [20]. Its mechanisms of targeting endothelial cells are distinct and Nm requires serum factors to bind to HUVECs [1], [2]. In these studies, the main serum factor involved was identified as vitronectin; fibronectin (Fn) also bound directly to Opc^+^ Nm, but to a lesser extent than Vn. This resulted in the localisation of the bacteria to endothelial RGD-recognising integrins particularly αvβ3 [1]. In contrast, the studies of Unkmeir *et al*. [4] demonstrated a prominent role of Fn but not Vn in Nm interactions with HBMECs. One notable observation made during our studies on HUVECs was that when using purified Vn, certain but not all preparations of Vn supported bacterial adhesion. As Vn may occur in a number of conformational forms [21], [22], [23], we hypothesised that Opc may only bind efficiently to certain conformational forms of Vn. In this regard, Vn is believed to undergo ligand-induced conformational change to exert its role in key processes of coagulation, fibrinolysis and in the protection of bystander cells against the terminal complement membrane attack complex (MAC) [23]; the processes of particular importance during Gram negative bacterial sepsis [24], [25]. Normally, Vn exists in its closed native conformation (depicted in summary final figure) [23], [26], [27] which has been shown to undergo conformational change on its activation by some of its physiological ligands which include, thrombin-antithrombin complex (TAT) and MAC. In one study, these were shown to be elevated during meningococcal sepsis [28]. Vitronectin conformational activation reveals a number of cryptic epitopes including the full exposure of the heparin binding site at the Vn C-terminal domain and the tyrosine sulphated residues in the connecting region (CR) of Vn (Y-S, final figure). Notably, two tyrosines in this region have been shown unequivocally to be sulphated in secreted vitronectin [29], [30], [31]. The active or unfolded Vn conformation could also be more favourable for cellular targeting via the cell binding domain (RGD) [27], [32]. In quantitative terms, Vn in fresh plasma is present at ∼300 µg/ml in its native conformation. However, ∼2% may be in its conformationally active form. This activated Vn concentration increases during blood coagulation to ∼7% [22], [23], [33].

The principal aim of our study described here was to assess the molecular mechanisms of Opc interactions with Vn and to examine the potential roles of Vn and Fn in Nm adhesion and invasion of human endothelial cells. Our studies have shown that Nm Opc can interact with Vn directly but its efficient interactions can only be shown with activated Vn. We have also demonstrated a novel mechanism of bacterial adhesion to human Vn. This involves direct binding to the Vn tyrosine sulphated region in which the sulphate moieties are necessary for bacterial adhesion. This mechanism may also be used in binding to vitronectin from other sources (bovine, murine). We further present studies comparing direct and indirect binding of Opc^+^ Nm to activated Vn, the latter occurs via heparin. By uncovering the underlying mechanisms of Opc/Vn interactions, this study additionally provides robust evidence and a rationale for the previous apparently divergent reports regarding the roles of serum proteins, especially Vn, in supporting meningococcal interactions with human endothelial integrins and subsequent cellular invasion.

The studies on Vn in relation to Nm seem pertinent also; as notably, meningococcal dissemination is associated with activation of coagulation, fibrinolysis and complement systems; the processes in which vitronectin is intimately involved as a scavenging factor for their end-products [24], [25], [28]. Accordingly, during meningococcal sepsis, consumption of plasma Vn has been shown to occur due to its widespread activation and subsequent removal from the circulation [28]. This would suggest that during intravascular spread, meningococci are likely to encounter a continuous supply of activated Vn in circulation.

Our current studies have employed acapsulate Nm isolates of serogroup A strain C751, which have been characterised extensively and used in a number of previous studies on meningococcal-endothelial interactions in order to unequivocally define the role of the Opc protein [1], [3], [10], [34]. In addition, to evaluate the role of vitronectin during infection, we have also used capsulate isolates of a serogroup B strain MC58 representing a phenotype that may be isolated from the blood (replete with capsule, pili, Opa and Opc).

## Results

### Relative efficacy of vitronectin and fibronectin in binding to Opc-expressing Nm and supporting adhesion and invasion of human endothelial cells

Previous studies have assigned different serum proteins as bridging molecules involved in mediating Nm Opc-dependent interactions with human endothelial cells of distinct origins: Vn for human umbilical vein endothelial cells (HUVECs) and Fn (but not Vn) for human brain microvascular endothelial cells (HBMECs) [1], [2], [4].

**Figure 1:**
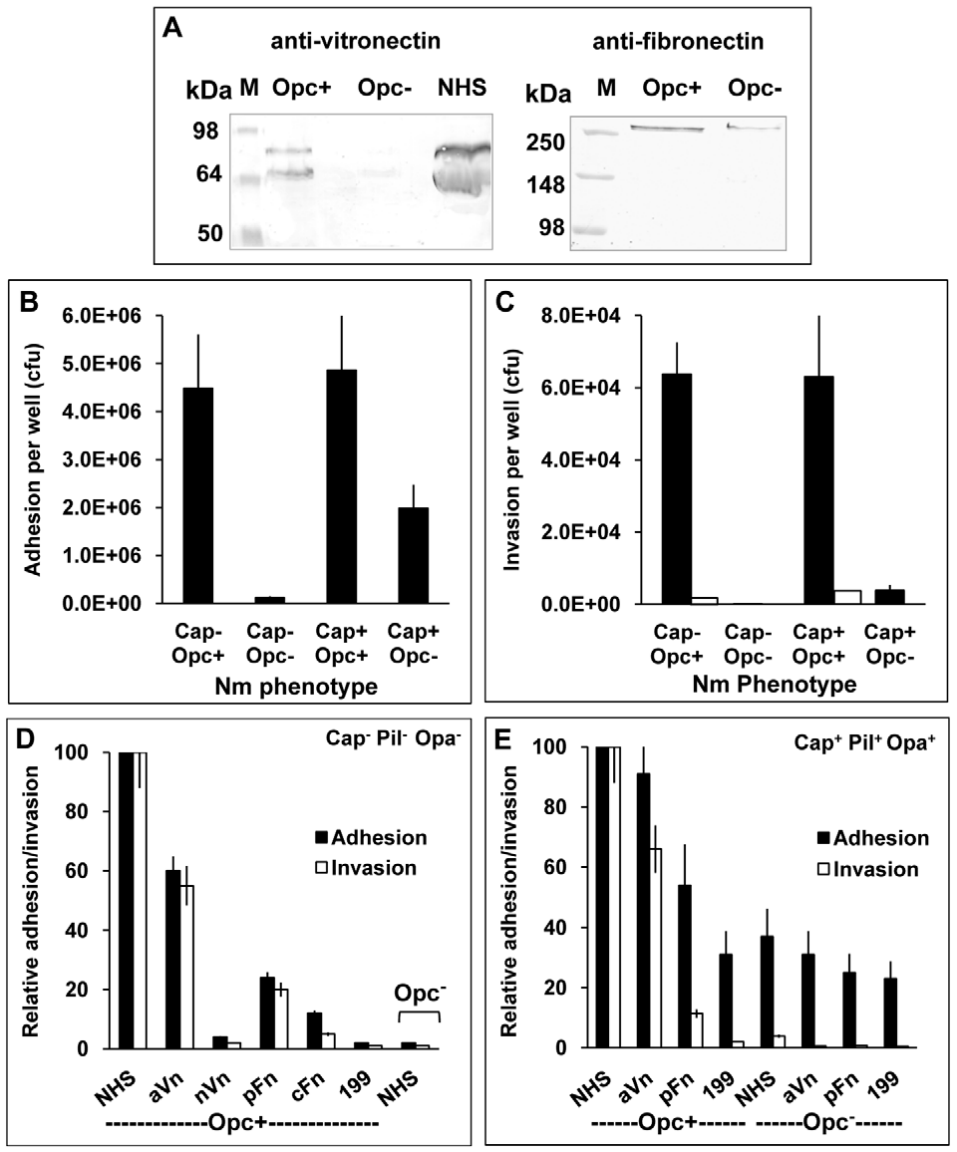
Invasion of HBMECs by capsulate and acapsulate meningococci is dependent on Opc-expression and serum components. (A) To determine the relative binding of the serum proteins to Opc^+^ and Opc^−^ *N. meningitidis*, bacteria were incubated with 10% normal human serum (NHS). The binding of vitronectin (Vn) and fibronectin (Fn) was assessed by applying lysates of serum coated, washed bacteria to SDS-polyacrylamide gels and western blotting. Anti-Vn and anti-Fn antibodies followed by AP-conjugated secondary antibodies were used to detect the proteins. Opc-expressing Nm bound to Vn and Fn, whereas Opc-deficient bacteria bound considerably weakly to the proteins. Lane marked NHS was loaded with the serum sample. The two vitronectin bands shown in the left blot correspond to the two molecular forms of vitronectin (75 and 65 kDa) found in human blood, the lower molecular form results from endogenous cleavage of a small region at the C-terminal end of some Vn molecules (see reference 26). (B, C) Relative efficiencies of Opc^+^ and Opc^−^ Nm isolates of strain C751 and MC58 (without or with capsule, pili and Opa expression respectively) in mediating HBMEC adhesion and invasion in serum supplemented media were determined by viable count and gentamicin protection assays. To confirm that gentamicin-resistant bacteria were truly internalised, cytochalasin D (CD) was used in some tests to prevent bacterial uptake into cells. In this case, no bacteria survived the gentamicin treatment (invasion levels indicated by blank columns in C) [35]. (D, E) In similar experiments, various purified serum components were compared with NHS in their ability to support HBMEC adhesion and invasion by acapsulate and capsulate meningococci. When supplemented, the infection media contained 10% NHS or 10 µg/ml of one of the following serum proteins: activated Vn (aVn), native Vn (nVn), cellular Fn (cFn) and plasma Fn (pFn), as in preliminary experiments, these were found to be the optimum concentrations required. The most effective serum component was found to be aVn in enhancing both cellular adhesion as well as invasion. Plasma Fn also supported adhesion and invasion but to a significantly lesser extent (D and E).

To clarify the adhesion supporting roles and mechanisms of Opc targeting of serum components, in the current studies, the roles of the serum proteins were further examined. Initially, the ability of Opc^+^ and Opc^−^ isolates to acquire serum factors from normal human serum was demonstrated by the exposure of Opc^+^ and Opc^−^ variants of strain C751 to normal human serum (NHS), followed by western blotting of adsorbed bacteria. Using anti-Vn and anti-Fn antibodies to overlay the blots, direct binding of Opc^+^ Nm to both these serum proteins could be seen; little or no binding was observed with Opc-deficient bacteria (Figure 1A).

In further experiments, the relative abilities of acapsulate Nm strain C751 and capsulate MC58 to attach to and invade HBMECs in the presence of NHS were compared. Several features were apparent from these studies. In serum supplemented medium, the strain C751 isolate (acapsulate and lacking pili and Opa adhesins) required Opc for cellular adhesion and invasion (Figures 1B and C). Whereas, capsulate MC58 (with pili and Opa expression), did not require Opc for a significant level of cellular adhesion. However, the Opc-deficient isolate of MC58 was not significantly invasive. In this strain however, Opc expression conferred both increased adhesion and invasion properties to the capsulate bacteria (Figures 1B and 1C). Cytochalasin D controls were used to confirm cellular invasion as assessed by gentamicin protection assays [35] (Figure 1C).

To assess the efficacy of different serum factors in supporting cellular adhesion and invasion of the above phenotypes, purified serum factors were used to supplement the infection media. We compared the native, folded Vn (nVn) as well as conformationally altered activated Vn (aVn) in addition to cellular and plasma forms of Fn (cFn, pFn). Of the purified proteins, the most efficient interaction was observed with aVn followed by pFn and then cFn. No significant cellular interactions were observed with nVn or unsupplemented media (Figure 1D shows the full analysis for Opc-expressing C751 isolate). For the capsulate piliated MC58, similar overall results were obtained. In Figure 1E, adhesion and invasion levels with the most effective serum components are shown for MC58. As in Figure 1B, a basal level of adhesion was observed for MC58 derivative even in the absence of Opc expression and was largely unaffected by the media supplements; but notably, no significant invasion was seen. The basal level of adhesion of the Opc^+^ isolate in the unsupplemented medium and that of Opc^−^ isolate under all the conditions tested is largely due to the pilus expression (see discussion). From these studies it is noteworthy that in a physiologically relevant meningococcal phenotype (MC58 phenotype used here), the subcapsular adhesin Opc plays an important role in endothelial cell invasion; it also enhances cellular adhesion. Both these functions appear to be largely dependent on the activated form of serum vitronectin.

### Preferential interactions of Opc^+^ *N. meningitidis* with activated vitronectin

#### (a) Activated Vn is depleted from serum on sequential incubation with Opc^+^ Nm

**Figure 2:**
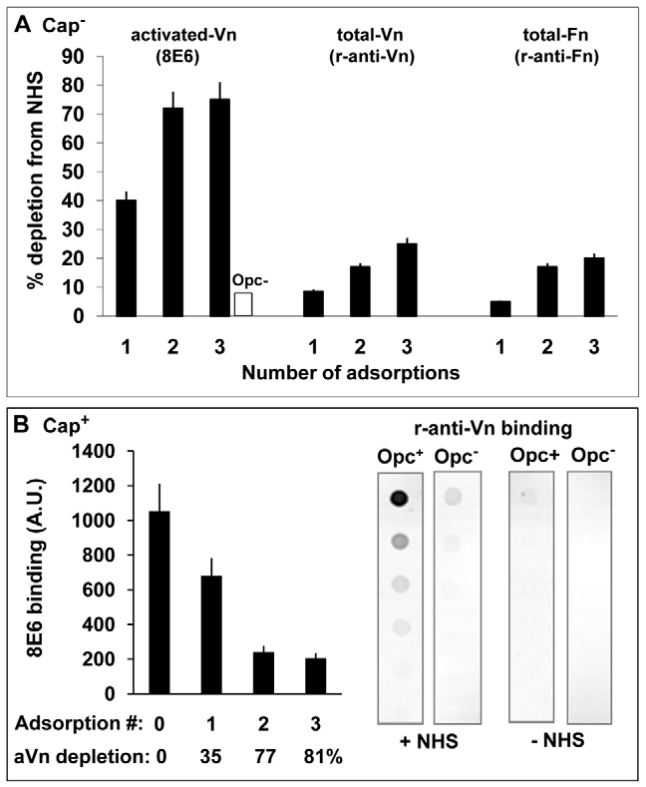
Activated vitronectin is adsorbed preferentially from serum by capsulate and acapsulate Opc-expressing *N. meningitidis*. (A) To assess which serum protein is preferentially removed from serum by Opc^+^ Nm, a sample of normal human serum was adsorbed three times sequentially with Opc-expressing phenotype of acapsulate C751. At each stage, aliquots of the adsorbed serum samples were retained. Unadsorbed serum and adsorbed samples were analysed by immuno-dot blot assay (followed by densitometric analysis) for activated and total Vn/Fn levels using the mAb 8E6 and the polyclonal rabbit anti-Vn or anti-fibronectin antibodies. The levels of each of these components extracted from serum by Opc^+^ Nm as a percent of the total present at the start of extraction are shown. In control experiments, in which Opc^−^ Nm were used, 8E6 binding to 3 x adsorbed serum did not alter significantly and amounted to less than 10% loss of aVn (blank column). (B) In a similar experiment, a sample of NHS was adsorbed sequentially with capsulate MC58 Opc^+^ and Opc^−^ phenotypes. The Opc^+^ bacteria effectively removed a large portion of aVn as assessed by mAb 8E6 binding to serum samples before and after incubation with Nm (B, left). Specific removal of Vn from NHS by Opc^+^ but not Opc^−^ MC58 isolates is apparent from the analysis of serum-adsorbed bacterial pellets which show that bound Vn was only significantly detectable on Opc^+^ bacteria that were exposed to NHS (B, right).

To assess the relative preference of Opc^+^ Nm for aVn, nVn and Fn when present simultaneously in normal human serum, a serum sample was incubated sequentially with three fresh suspensions of acapsulate Opc^+^ C751 isolate. Aliquots of the serum after each extraction were analysed for remaining levels of the proteins by immuno-dot blotting (Figure 2A). Total Fn and total Vn levels were determined by the use of polyclonal antibodies. As activated/unfolded human Vn has been shown to reveal cryptic epitopes, one of which is recognised by the monoclonal antibody (mAb) 8E6, this conformation-dependent antibody was used specifically to detect aVn in serum [22], [36]. The results illustrated that the levels of activated Vn decreased in NHS dramatically by over 75% with two sequential Opc^+^ bacterial incubations but a relatively small overall depletion of total Vn or Fn levels (25 and 20% respectively) occurred even after three extractions (Figure 2A). Less than 10% of 8E6-binding component was lost from serum with Opc-deficient bacteria (Figure 2A).

In assessing the level of decrease of the serum proteins, it is pertinent to note that activated Vn in whole serum may amount to over 7% [33]. However, in decomplemented serum, which is subjected to heat treatment, additional conversion of nVn to aVn will occur [22], [37]. In two separate experiments, we observed the level of aVn in serum to increase by 2-3-fold after heat treatment as determined by 8E6 binding, thus approaching the levels of total Vn lost (20–25%) after Opc^+^ bacterial incubation (Figure 2A).

In complementary experiments, removal of heparin-binding components from human serum, which removed activated Vn but not native Vn, almost totally abrogated serum-dependent Nm interactions (not shown).

To assess capsulate bacterial interactions with serum vitronectin, freshly prepared viable suspensions of Opc^+^ and Opc^−^ derivatives of MC58 were incubated with NHS. Opc^+^ phenotype was again effective in removing activated Vn which was depleted by 81% after 3 adsorption cycles (Figure 2B, left). Very little 8E6-binding component was lost from serum with Opc-deficient MC58 (not shown). In agreement with these observations, when the adsorbed bacterial pellets were examined for bound Vn using a polyclonal Vn antibody in a immuno-dot blot assay, the antibody only reacted with Opc^+^ but not Opc^−^ MC58 that had been exposed to NHS (Figure 2B, right).

Overall, the following conclusions can be drawn from the data presented on the examined serum proteins extracted by Opc^+^ and Opc^−^ Nm (Figure 1A and 2): a) that Vn as well as Fn from human serum bind to Opc^+^ Nm (Figure 1A); b) the binding of serum Vn is facilitated by the expression of Opc (immunoblots in Figures 1 and 2); c) after three sequential extractions with Opc^+^ Nm, the decrease in total Vn levels roughly equal the levels of aVn present in the decomplemented NHS used. These observations are consistent with the notion that aVn is extracted in preference to nVn. This preference for aVn was further investigated in direct binding assays.

#### (b) Denaturation of native Vn by heat treatment increases its recognition by Opc-expressing Nm

**Figure 3:**
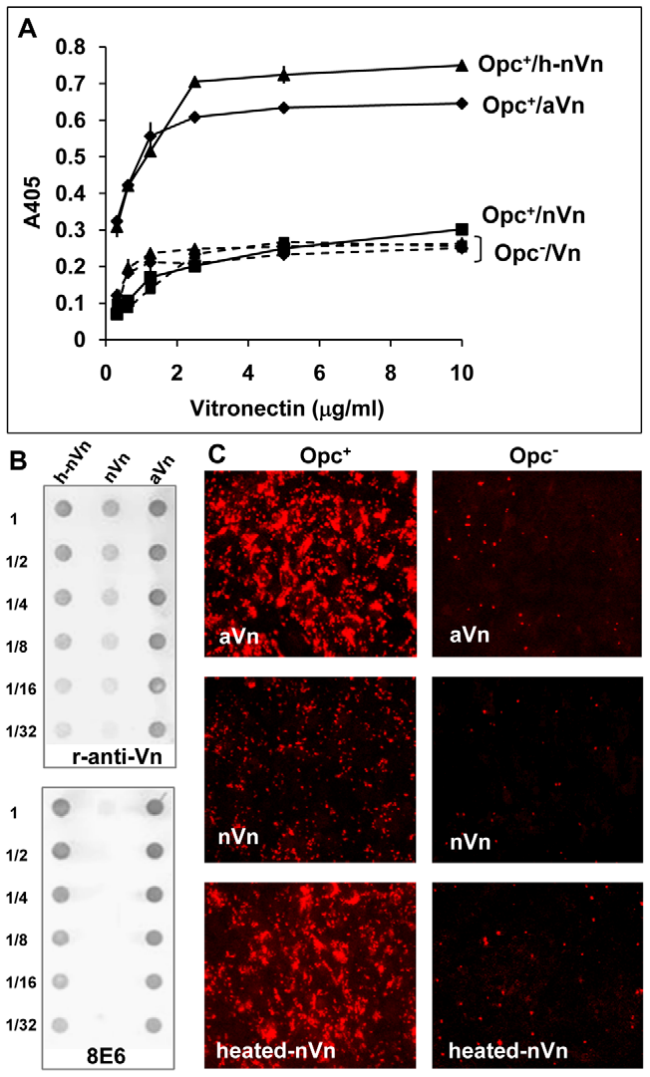
Deliberate modification of nVn conformation increases Opc–expressing meningococcal interactions with Vn and with HBMECs. (A) Purified native Vn (nVn) was subjected to limited heat denaturation to relax its folded conformation and reveal the mAb 8E6 binding site. To assess the levels of binding of Opc^+^ Nm to nVn, heated nVn (h-nVn) and the serum-derived heparin-purified activated Vn preparation (mAb 8E6 binding form), these proteins were overlaid on to immobilised Opc^+^ and Opc^−^ isolates of strain C751 and Vn binding determined using polyclonal anti-Vn antibody. As bacteria lacking Opc exhibited the same level of binding to all vitronectin preparations, these are labelled as (Opc^−^/Vn broken lines). (B) The vitronectin samples used for ELISA were also analysed for their activation status using the mAb 8E6 and the polyclonal anti-Vn antibody (r-anti-Vn) by immuno-dot blotting. The conformation-dependent mAb 8E6 only reacted with aVn and heated nVn demonstrating the exposure of its epitope on heat treatment of nVn. Rabbit antibodies also bound more effectively to the same two forms of Vn suggesting that aVn and h-nVn present larger numbers of epitopes. (C) Immunofluorescence analysis was performed using confluent HBMECs infected with Opc^+^ and Opc^−^ C751 isolates in media supplemented with aVn, nVn and heated nVn illustrating their relative abilities in supporting bacterial adhesion. Bacteria were labelled using anti-Nm antiserum and rhodamine-conjugated secondary antibody.

To establish further the importance of Vn conformation, purified preparations of native Vn and activated Vn were used to assess Nm Opc interactions in a quantitative ELISA. As adsorption to plastic has been shown to denature native Vn to variable extents [21], [38], the studies were carried out using bacteria in solid phase and Vn preparations in fluid phase. Compared to aVn, nVn bound poorly to Opc^+^ bacteria; Opc^−^ variant of the same strain did not bind well to either preparation (Figure 3A). As noted above, mild heat treatment of nVn has been shown to unfold the protein and increase its access to the mAb 8E6 [36], [37]. When nVn was subjected to such heat treatment, mAb 8E6 binding increased (Figure 3B, lower panel) and concurrently Opc^+^ (but not Opc^−^) meningococcal interactions increased substantially (Figure 3A). The data confirm that 8E6 binding and bacterial interactions both require similar Vn conformations. In addition, when nVn, heated-nVn and aVn were examined for their adhesion supporting activities, the heated-nVn behaved in a similar manner to aVn, both supporting substantial Opc^+^ Nm binding to HBMECs whereas nVn was ineffective (Figure 3C).

Taken together, the above data strongly support the notion that activated Vn is the preferred form of Vn for Opc interactions. In further studies described below, we sought to define the molecular basis of the direct interactions of Opc with human serum Vn.

### Opc interactions with aVn are inhibited by the conformation-dependent mAb 8E6

**Figure 4:**
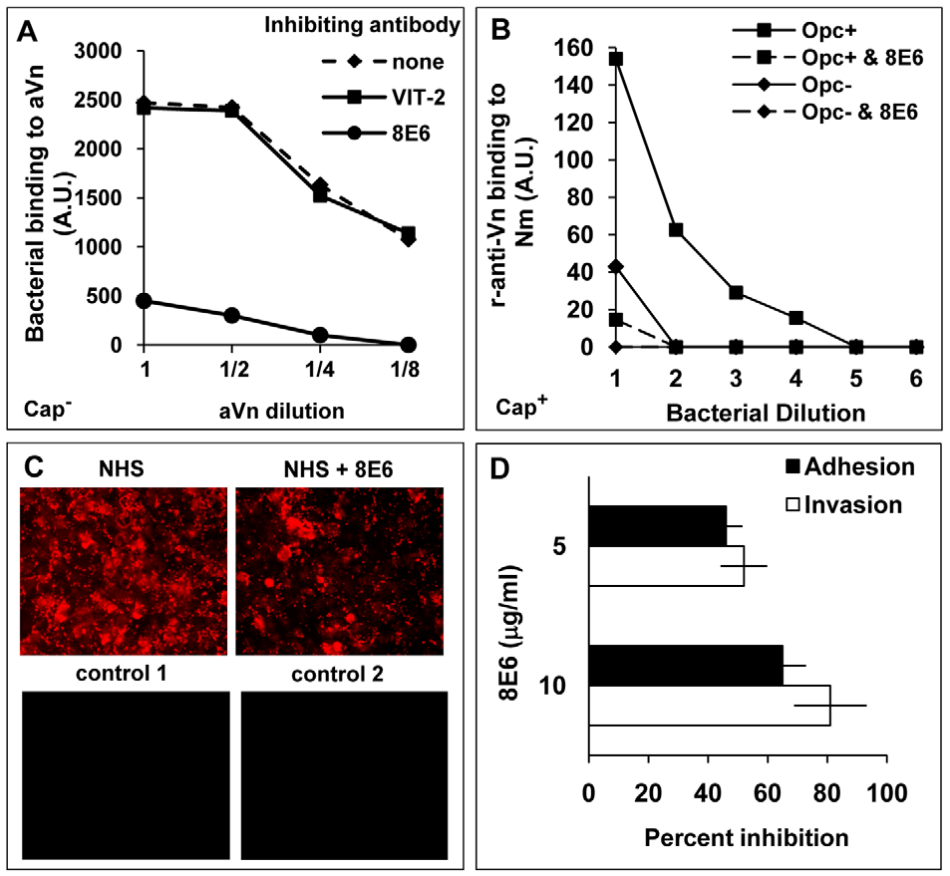
Monoclonal antibody 8E6 against a cryptic epitope of vitronectin inhibits Opc-mediated interactions of *N. meningitidis*. (A) The ability of anti-Vn antibodies 8E6 and VIT-2 to inhibit bacterial binding to immobilised aVn was investigated by immuno-dot blot assays in the presence or absence of the anti-Vn antibodies added at 10 µg/ml prior to Opc^+^ acapsulate Nm additions. Relative levels of Opc^+^ Nm binding is shown as arbitrary units determined by densitometric analysis as described in the text. 8E6 but not VIT-2 inhibited direct bacterial binding to purified aVn. (B) 8E6 also prevented adsorption of aVn on to capsulate Opc^+^ bacteria from NHS; the procedure was carried out as described in the legend to figure 2. The mAb 8E6 when present was used at 10 µg/ml. (C, D) The effect of 8E6 on adhesion/invasion of HBMEC by acapsulate Opc^+^ bacteria in the presence of 10% NHS was assessed using immunofluorescence adhesion assay (C) or viable count assays (D) performed as described in methods. Bacteria in (C) were labelled using anti-Nm antiserum and rhodamine-conjugated secondary antibody. For controls, either an unrelated primary antibody (control 1) or secondary antibody only (control 2) were used. As addition of 8E6 results in a significant and a dose-dependent inhibition of adhesion and invasion (D), activated Vn appears to be one of the main serum factors supporting Nm interactions with human endothelial cells.

To identify the binding site of Opc on activated Vn, initially we investigated if the mAb 8E6 could inhibit Opc-expressing Nm binding to the activated Vn by immuno-dot blot assays. Surprisingly, the antibody almost totally inhibited Opc^+^ Nm binding to aVn (Figure 4A) and prevented aVn adsorption from NHS on to Opc^+^ capsulate meningococcal surface (Figure 4B). Very low levels of Vn adsorbed on to some Opc^−^ cultures could be due to Opc^+^ phase variants that naturally arise or due to non-specific adsorption of Vn on bacterial surface; although, the data do not rule out the possibility of Vn binding to other bacterial component/s. However, the observed difference between Opc^+^ and Opc^−^ bacteria is substantial, and clearly significant. In addition, 8E6 significantly inhibited serum-mediated Opc^+^ Nm adhesion and invasion of HBMECs (Figure 4C and 4D). Several conclusions can be drawn from these observations: that Nm can bind to activated Vn at a site overlapping or closely positioned to the mAb 8E6 binding site, that activated Vn recognised by 8E6 may represent a significant portion of the serum component involved in mediating Nm interactions with HBMECs (Figure 4D) and that other serum components may also participate in supporting bacterial binding to human endothelial cells but to a relatively lesser extent than activated Vn.

### Identification of the vitronectin region involved in binding to *N. meningitidis* Opc

**Figure 5:**
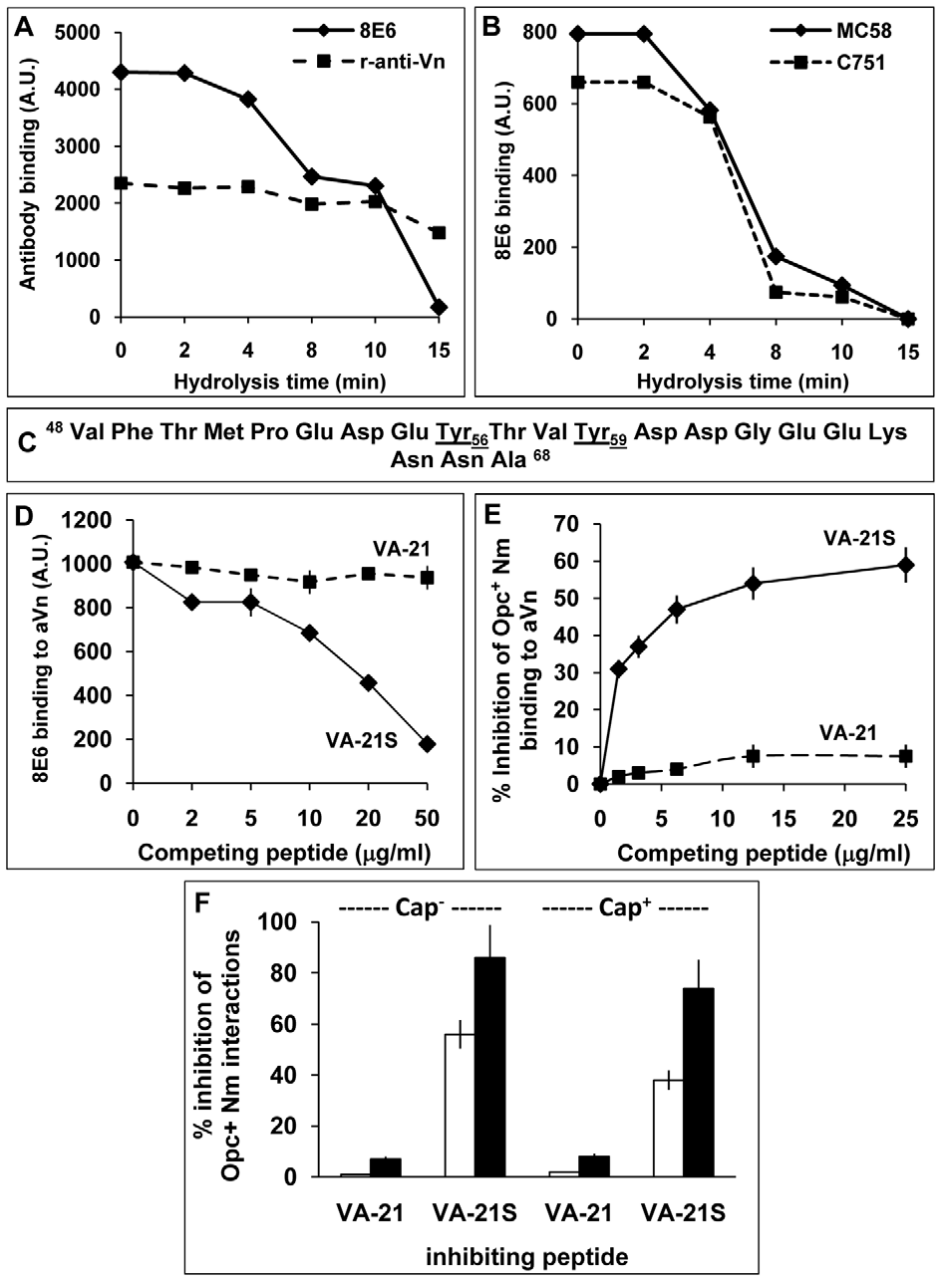
The nature of the vitronectin epitope recognised by *N. meningitidis* Opc. (A, B) **Acid hydrolysis of vitronectin.** Immuno-dot blot assays were performed using acid-treated (1 M HCl, 80°C), immobilised aVn on to nitrocellulose strips. The strips were blocked and overlaid with antibodies or bacteria and analysed for the ability of Vn to retain binding of the mAb 8E6 and polyclonal rabbit anti-Vn antiserum (A) or acapsulate (C751) and capsulate (MC58) Opc^+^ Nm (B). Binding of bacteria and 8E6 declined simultaneously during acid hydrolysis of Vn known to hydrolyse the sulphate groups. (C–E) **Studies using synthetic vitronectin peptides.** (C) Sequence of synthetic peptides corresponding to the residues 48–68 containing the 8E6 binding site of vitronectin. The peptide VA-21 was synthesised in a non-sulphated form whereas the peptide named VA-21S was sulphated at residues 56 and 59 as in vitronectin. (D) In a competition immuno-dot blot assay, the ability of the mAb 8E6 to bind to immobilised aVn in the absence or presence of the peptides was assessed. 8E6 binding was quantified using AP-conjugated rabbit anti-mouse secondary antibody followed by densitometry. VA-21S but not VA-21 inhibited 8E6 binding to immobilised aVn. (E) In an ELISA, the binding of acapsulate C751 Opc^+^ Nm overlaid on to immobilised aVn was inhibited significantly by the addition of increasing concentration of synthetic peptide VA-21S but to a very low extent with the equivalent unsulphated peptide VA-21. (F) To assess the efficacy of VA-21 and VA-21S to inhibit aVn-mediated adhesion and invasion by acapsulate and capsulate Opc^+^ Nm, viable count experiments were performed. VA-21 or VA-21S peptides (25 µg/ml) were used to pre-coat meningococci (15 min. incubation) which were then added to HBMEC monolayers in infection medium supplemented with aVn (10 µg/ml). VA-21 had no significant effect on adhesion (blank columns) or invasion (filled columns). In contrast, VA-21S reduced cellular adhesion and invasion significantly with a more dramatic effect on invasion levels of both strains.

Relatively mild acid hydrolysis of Vn using conditions that destroy the labile O-linked sulphate groups has been reported to diminish the binding of the mAb 8E6 [30]. This mAb has been shown to require sulphation of the tyrosine residues (Y_56_ and Y_59_) of the connecting region of Vn for binding [30], [31]. Accordingly, Vn expressed in *E. coli* does not bind to 8E6 [22]. To assess if bacterial binding is also affected by such acid treatment of Vn, aVn samples treated at 80°C with 1 M HCl for increasing time periods (as described in methods) were dotted on to nitrocellulose and overlaid with either 8E6, polyclonal anti-Vn antibody or Opc^+^ Nm. The polyclonal antibody was used to assess if the overall vitronectin structure is affected by the acid treatment. The mAb 8E6 binding and Opc-expressing bacterial interactions (both acapsulate C751 and capsulate MC58) with Vn declined in concert, and no binding of either could be detected when Vn was treated for >15 min (Figures 5A and 5B). In contrast to 8E6, polyclonal anti-Vn antibody still bound to Vn significantly at the end of acid hydrolysis (Figure 5A).

Overall, these data suggest that the binding sites of the mAb 8E6 and Nm Opc share similar characteristics and may be related to the sulphation of tyrosines at sites 56 and 59. However, it could be argued that acid hydrolysis may also destroy other specific epitopes not detected by the polyclonal antibody. Therefore, to further assess if tyrosine sulphation is required for bacterial binding, we used sulphated and unsulphated peptides spanning the Vn region 48–68 (Figure 5C), termed VA-21S and VA-21 respectively. These peptides otherwise carried no further modifications. As the mAb 8E6 binding requires sulphated tyrosines, only VA-21S would be expected to bind to the antibody and prevent its binding to aVn. Accordingly, in competitive assays, the binding of the mAb 8E6 to the whole immobilised aVn sample was prevented in the presence of added competing VA-21S but not with VA-21 (Figure 5D). Similarly, Opc^+^ Nm binding to aVn was also specifically and significantly inhibited by VA-21S peptide in a concentration dependent manner but not by VA-21 (Figure 5E). The data demonstrate that Nm Opc interactions with Vn involve an epitope within the CR residues 48–68 and require sulphation of tyrosines for effective interactions. The two peptides were also assessed in their ability to inhibit aVn-dependent bacterial binding and invasion of HBMECs. VA-21S, but not VA-21, inhibited Opc^+^ Nm adhesion to the endothelial cells, it also dramatically inhibited invasion of HBMECs by both capsulate and acapsulate Nm (Figure 5F).

### Demonstration of the direct binding of Opc^+^ *N. meningitidis* to sulphated but not phosphorylated vitronectin peptides spanning the residues 43–68

**Figure 6:**
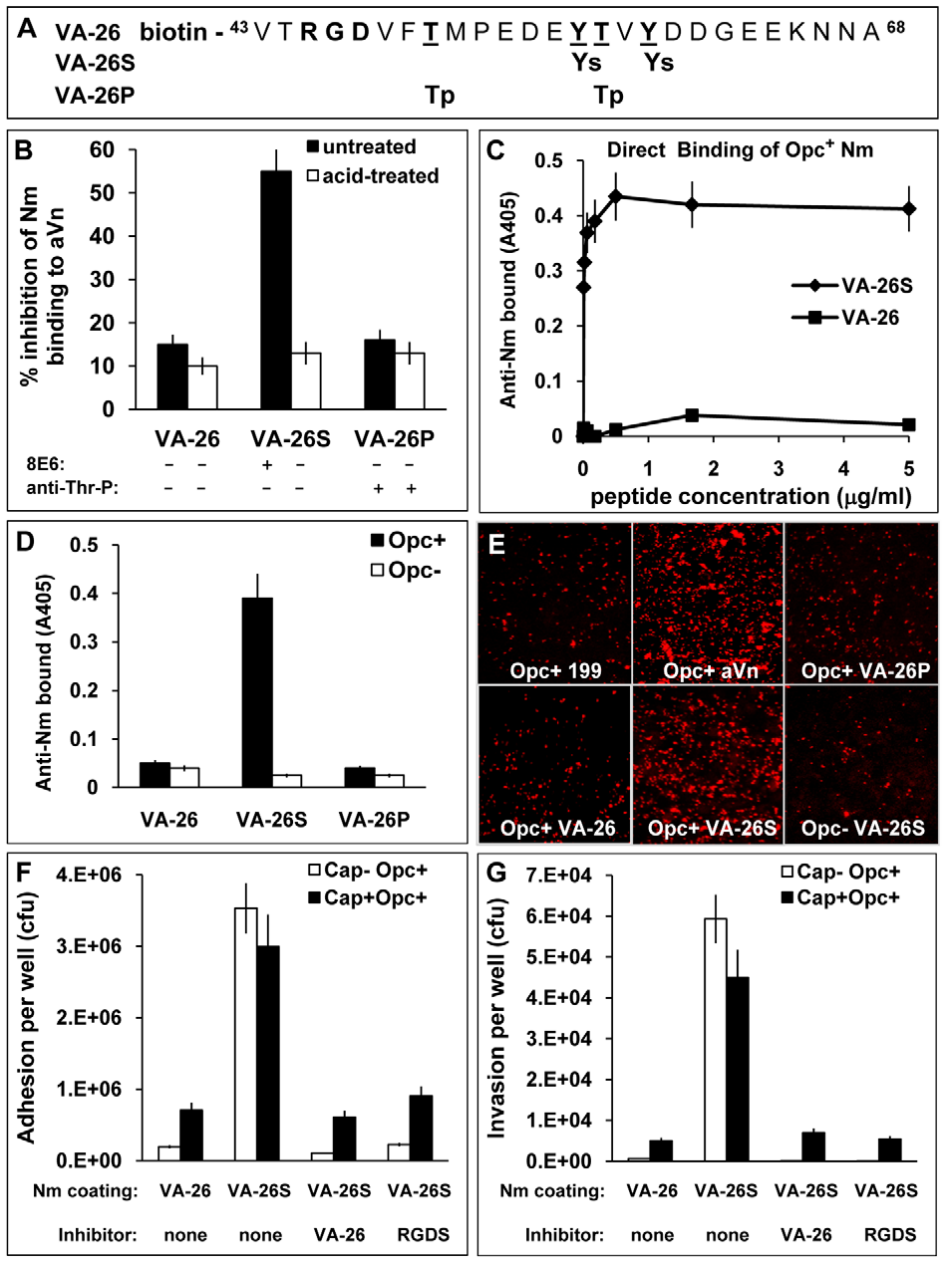
The VA-26S sulphated peptide contains the features required and sufficient for Opc binding and cellular invasion. (A) Structures of the biotinylated peptides spanning the Vn region 43–68. VA-26 peptide was unmodified other than at the N-terminal residue which was linked to biotin. Additionally, VA-26S contained sulphated Y_56_ and Y_59_ and VA-26P contained phosphorylated T_50_ and T_57_. (B) Using 25 µg/ml each of untreated and prior acid-hydrolysed peptides, their relative abilities to inhibit C751 Opc^+^ bacterial interactions with immobilised aVn were examined by ELISA. Only untreated VA-26S inhibited bacterial binding to aVn. The table summarises the effect of acid-treatment on the peptides (see Figure S1), only the sulphated residues are hydrolysed and consequently only the mAb 8E6 binding to VA-26S is affected after treatment. (C) To examine the direct binding of Opc^+^ Nm of strain C751 to VA-26S and VA-26, the peptides were first immobilised on extravidin-containing plates (Figure S2). Bacterial binding was detected using anti-Nm antiserum. Opc^+^ Nm bound significantly only to VA-26S. (D) Relative levels of direct binding of Opc^+^ and Opc^−^ Nm to immobilised VA-26, VA-26S and VA-26P peptides by ELISA. In each case, saturating levels of peptides were used (5 µg/ml each, Figure S2). Bacterial binding as detected using anti-Nm antiserum shows only VA-26S and Opc^+^ Nm interactions. (E) Immunofluorescence analysis of the ability of VA-26 peptides, which contain the integrin-binding domain RGD, to mediate bacterial binding to HBMECs. Opc^+^ bacteria but not Opc^−^ bacteria localised on to HBMECs in significantly higher numbers in the presence of VA-26S (and aVn control) but not with other peptides (all used at 20 µg/ml). (F and G) To assess the efficacy of VA-26S in mediating acapsulate and capsulate bacterial adhesion and invasion, viable count experiments were performed using VA-26 or VA-26S peptide-coated bacteria. In some experiments VA-26 (20 µg/ml) and RGDS (0.25 mM) were used to preincubate HBMECs to act as inhibitors of RGD-mediated binding of VA-26S-coated bacteria. The data illustrate that two key features (sulphated tyrosines and RGD) present in VA-26S are required and sufficient for mediating bacterial invasion of HBMECs.

As the 21-mer peptides (VA-21/S) used above proved to be resistant to immobilisation on a variety of solid surfaces, further experiments were carried out on biotin-tagged 26-mer peptides spanning the Vn region 43-68 (Figure 6A). It is noteworthy that these peptides do not contain any of the heparin binding domains of Vn (see final figure). In addition to the unmodified peptide (VA-26), both sulphated (VA-26S with Tyr_56_S, Tyr_59_S) as well as phosphorylated (VA-26P with Thr_50_P, Thr_57_P) were derived. The latter peptide design was chosen as protein kinase CK2 (casein kinase 2) has been reported to phosphorylate T_50_ and T_57_ of Vn extracellularly [39], [40], [41].

Initially, we compared the effects of the three peptides in a competitive ELISA to assess their ability to inhibit Opc^+^ Nm binding to immobilised aVn. In addition, we also subjected the peptides to mild acid-hydrolysis to remove the sulphate residues from VA-26S. Such a treatment (<20 minutes in 1 M HCl at 80°C) was not expected to affect the phosphorylation of the VA-26P peptide as prolonged hydrolysis >1.5 h in 6 M HCl at 110°C in vacuum has been shown to be required to extract intact phosphothreonines and phosphoserines from proteins [42], [43]. Accordingly, mAb 8E6 binding was abrogated but the phosphate residues on VA-26P were not at all affected by acid-treatment and the anti-phosphothreonine antibody binding remained unchanged (Figures 6B and S1). Concurrently, only the untreated VA-26S inhibited Opc^+^ Nm binding to aVn (Figure 6B). The data suggest that the VA-26S peptide behaved identically to the slightly shorter untagged peptide (Figure 5E) and that neither the phosphorylated nor the unmodified peptide could interfere with bacterial binding to aVn.

To assess if bacteria could bind directly to peptides, ELISA plates with immobilised extravidin were loaded with increasing concentrations of the biotinylated peptides. The peptide binding was demonstrated by using 8E6 and anti-Thr-P antibodies (Figure S2A). Binding of the unmodified peptide (VA-26) was determined by indirect ELISA in which extravidin plates were sequentially exposed to the biotinylated peptides VA-26 followed by VA-26S. The binding of VA-26S was then assessed by the use of mAb 8E6 (Figure S2B). When bacterial binding was examined to peptide-loaded plates, the level of Opc^+^ bacterial binding paralleled the VA-26S peptide-loading, reaching a saturation point at about 1 µg/ml peptide concentration used for immobilisation (Figures 6C and S2A). In addition, Opc^+^ bacterial adhesion was observed only when the plates contained VA-26S but not with VA-26 or VA-26P peptides (Figure 6D).

The above data provide compelling evidence that sulphated tyrosines of vitronectin create a binding epitope for Opc and show that negatively charged phosphorylated threonine residues do not substitute for sulphated tyrosine residues in Opc-mediated binding at the CR of Vn. Whether phosphate groups, when present in addition to sulphate groups, further strengthen the interactions of the synthetic peptides was not investigated.

### The sulphated peptide spanning the Vn residues 43–68 (VA-26S) contains features required for cellular adhesion and invasion

The VA-26 peptides with an RGD sequence upstream of the tyrosine sulphation site, as in the native Vn, were used to examine further the role of the Vn CR in Opc^+^ Nm targeting of endothelial integrins. Using these peptides, specific and significant binding of Opc^+^ bacteria to HBMECs in the presence of VA-26S but not the other peptides could be demonstrated (Figure 6E). In quantitative adhesion and invasion experiments, adhesion to and invasion of HBMECs by both capsulate and acapsulate meningococci was enhanced by VA-26S in a RGD and tyrosine-sulphation-dependent manner (Figure 6F and G); as both VA-26 peptide with an RGD sequence as well as RGDS peptides incubated with HBMECs prior to the addition of VA-26S precoated bacteria inhibited bacterial interactions. These studies highlight the sufficiency of sulphation of the tyrosines and an RGD sequence as presented in VA-26S for meningococcal invasion of human brain endothelial cells.

In complementary experiments, we also investigated the effect of VA-21 and VA-21S to inhibit VA-26S-mediated HBMEC interactions. In accordance with its effect on aVn-mediated cellular interactions (Figure 5F), dramatic inhibition of VA-26S-mediated adhesion and invasion occurred with VA-21S; VA-21 had no effect. With VA-21S, the inhibition of adhesion of C751 Opc^+^ Nm approached 80% and invasion 100%. For piliated capsulate Opc^+^ MC58, adhesion was inhibited less well (∼40%, as pili continue to assist in cellular adhesion) but invasion was inhibited by over 80% (data not illustrated).

### Species specificity of Opc and 8E6, and their requirements for binding to vitronectins

**Figure 7:**
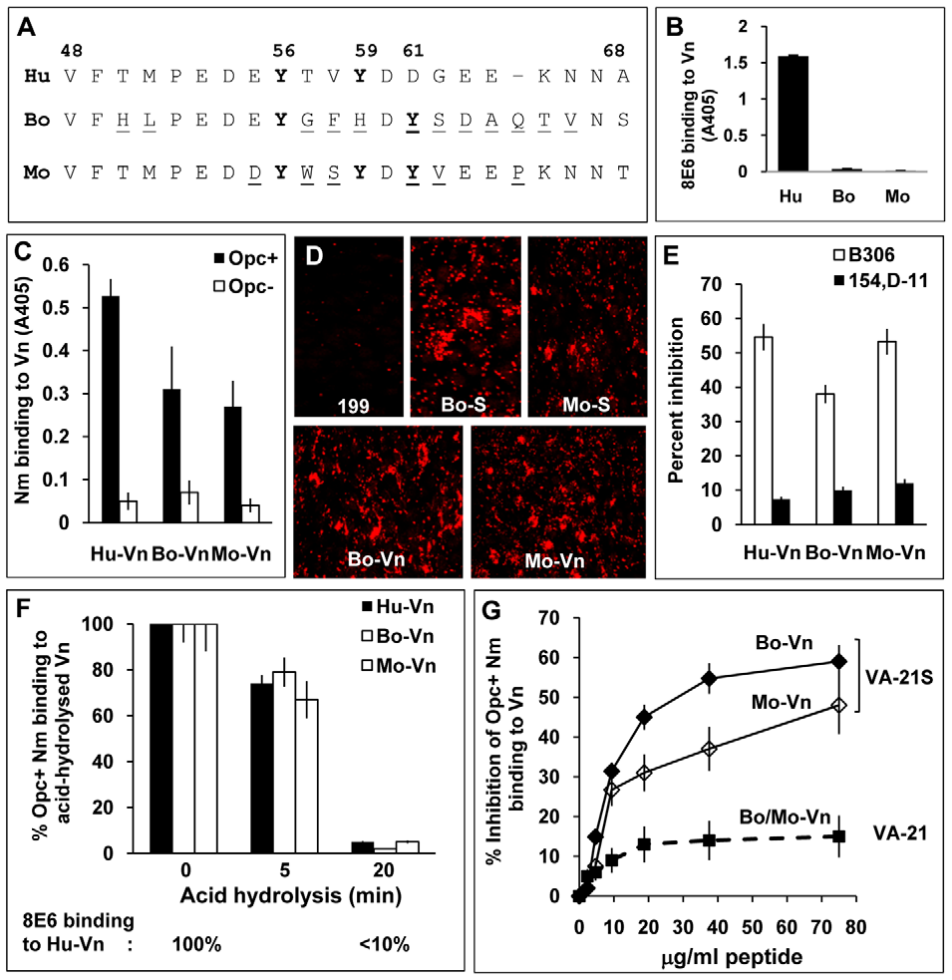
Species specificity of Opc and 8E6 and their binding requirements. (A) Sequences flanking the fully conserved Y_56_ in human (Hu), bovine (Bo) and murine (mouse, Mo) vitronectins aligned using MegAlign Lasergene software (DNASTAR). The variant residues in bovine and murine vitronectins are underlined. (B) Reactivity of the mAb 8E6 with immobilised vitronectins from distinct sources was analysed by ELISA demonstrating its specificity for human Vn. (C) In a similar experiment, the binding of C751 Opc^+^ and Opc^−^ Nm to immobilised vitronectins was assessed by bacterial overlay. Although human Vn was the most recognised, Opc^+^ Nm also bound to bovine and mouse Vn at significantly higher levels compared with Opc^−^ Nm. (D) The relative abilities of bovine and mouse sera (used at 10%) and purified vitronectins (10 µg/ml) to support C751 Opc^+^ Nm binding to HBMECs were assessed by immunofluorescence analysis as described in methods. (E) Two mAbs against Opc known to bind to loop 2 (B306) or loops 4/5 (154,D-11) were used in competition studies to assess their ability to block Opc/Vn interactions. Bacterial suspensions were pre-incubated with 30 µg/ml of the antibodies for 20 min prior to addition to Vn. B306 against loop 2 of Opc caused significantly higher inhibition of meningococcal binding to all three Vn samples. (F) The potential role/s of sulphated tyrosines in each of the vitronectins was assessed by acid hydrolysis as described in methods. The relative levels of bacterial binding to the animal vitronectins are shown as blank columns (bovine Vn: middle columns, mouse Vn: right columns) and to human Vn (used for comparison) as filled columns. Percent binding compared with untreated vitronectins demonstrates similar decline in bacterial binding to all three proteins concurrently with the decline in 8E6 binding (monitored simultaneously using human Vn shown below the graph). (G) In a competitive ELISA, VA-21S peptide but not VA-21 inhibited bacterial binding to immobilised bovine and murine vitronectins in a manner similar to human vitronectin suggesting similar mechanisms of targeting the three vitronectins.

As bovine serum is frequently used as a supplement in growth and infection media and mice are sometimes used for studies on systemic meningococcal infections, vitronectins of these animal origins relevant to Nm studies were examined for conservation of the 8E6/Nm binding sites. The relevant sequences of the human, bovine and murine vitronectins are shown in Figure 7A. Tyrosine at position 56 is conserved in all three vitronectins, and Y_59_ is present in human and murine but not bovine Vn. There are also significant differences in bovine and murine vitronectins especially in residues down-stream of Y_56_. Notably also, there is a tyrosine residue at position 61 in both bovine and murine Vn not present in human Vn. Bovine Vn also does not contain threonine residues at positions 50 and 57. Initially, the reactivity of the mAb 8E6 with immobilised vitronectins (all prepared using heparin-sepharose) from distinct sources was analysed by ELISA. Only human Vn was recognised by 8E6 (Figure 7B). In a previous study, efficient recognition of Vn peptides by 8E6 was shown to be dependent on sulphated Y_59_, although the maximum 8E6 binding occurred when both Y_56_ and Y_59_ were sulphated [30]. The three Vn samples were also subjected to limited heat treatment to unfold any closed conformers. This treatment did not alter the mAb 8E6 binding pattern (not shown).

To assess bacterial recognition of the serum proteins, immobilised Vn preparations were overlaid with Opc^+^ and Opc^−^ Nm in an ELISA. Opc^+^ bacteria bound to human Vn to the greatest extent but significant levels of binding to bovine and murine Vn (compared with Opc^−^ Nm) also occurred (Figure 7C). In accordance with 8E6 recognition of human but not other vitronectins, inclusion of 8E6 with Opc^+^ Nm in overlay experiments resulted in inhibition of bacterial binding to human Vn observed above (Figure 4A) but not to other vitronectins (data not shown). As with mAb 8E6 binding, prior heat treatment of the vitronectins did not alter the pattern of bacterial binding (not shown).

Bovine serum enhanced endothelial interactions of Nm has been reported previously [1]. Further, in accordance with Nm Opc binding to the animal vitronectins, both bovine and murine sera and purified vitronectins also supported bacterial binding to HBMECs (Figure 7D).

### Similarities of Opc interactions with human, bovine and murine vitronectins

The mAb B306 against an epitope on the loop 2 of Opc [44] has been shown previously to inhibit Nm Opc-mediated, Vn-dependent interactions with human endothelial cells [2], [3]. To assess if the direct binding to the various vitronectins can be inhibited similarly by this mAb, first an ELISA was performed using increasing concentrations of the mAb B306 and another mAb, 154,D-11, that binds to an epitope in loops 4/5 [44] to inhibit bacterial binding to aVn using immobilised bacteria. Concentration-dependent inhibition of binding of aVn to Opc^+^ Nm was observed in the presence of B306 but not with the mAb 154,D-11 (Figure S3). Similarly, Opc^+^ Nm binding to immobilised bovine and murine Vn was inhibited with B306 whereas 154,D-11 had significantly less inhibitory effect on bacterial binding (Figure 7E). Taken together, the data indicate that similar mechanisms may determine the direct binding of Opc to the three vitronectins.

To investigate if binding to bovine and murine Vn was dependent also on sulphated tyrosines, the Vn preparations were subjected to acid hydrolysis prior to the examination of Opc^+^ Nm binding by ELISA. In each case, acid hydrolysis for 20 min resulted in abrogation of bacterial binding (Figure 7F) consistent with the role of Y-S in bacterial binding. Further in a competition ELISA, VA-21S peptide significantly inhibited Opc^+^ Nm binding to immobilised bovine as well as murine Vn (Figure 7G). Thus overall, the data indicate somewhat variant requirements for Nm Opc and the mAb 8E6 binding to the human and animal vitronectins, however, sulphation of tyrosine appears to be important in each case.

### Dual mechanisms of interactions of *N. meningitidis* Opc protein with human vitronectin

Besides the sulphated tyrosines, Vn molecule has been shown to contain several heparin-binding domains (HBD), one of which, located in the C-terminal region spanning the residues 341–380 has been shown to mediate high affinity interactions with heparin (see schematic diagram final figure, HBD) [45]. As Opc is also a heparin-binding protein [20], that Opc may bind to the HBD of Vn via a heparin bridge is entirely possible; this has been implied from earlier studies [46]. To investigate the possible and relative roles of both the CR and the HBD of the activated Vn in Opc binding, we initially analysed if any heparin or heparin-like molecules become attached to bacteria grown on solid agar-containing media as bacterial acquisition of charged molecules could alter the observed effects of added heparin [47]. However, no differences were observed between C751 isolates grown on GC-agarose medium with highly purified agarose as a gelling agent (see Methods), or those grown on HBHI (Figure S4).

**Figure 8:**
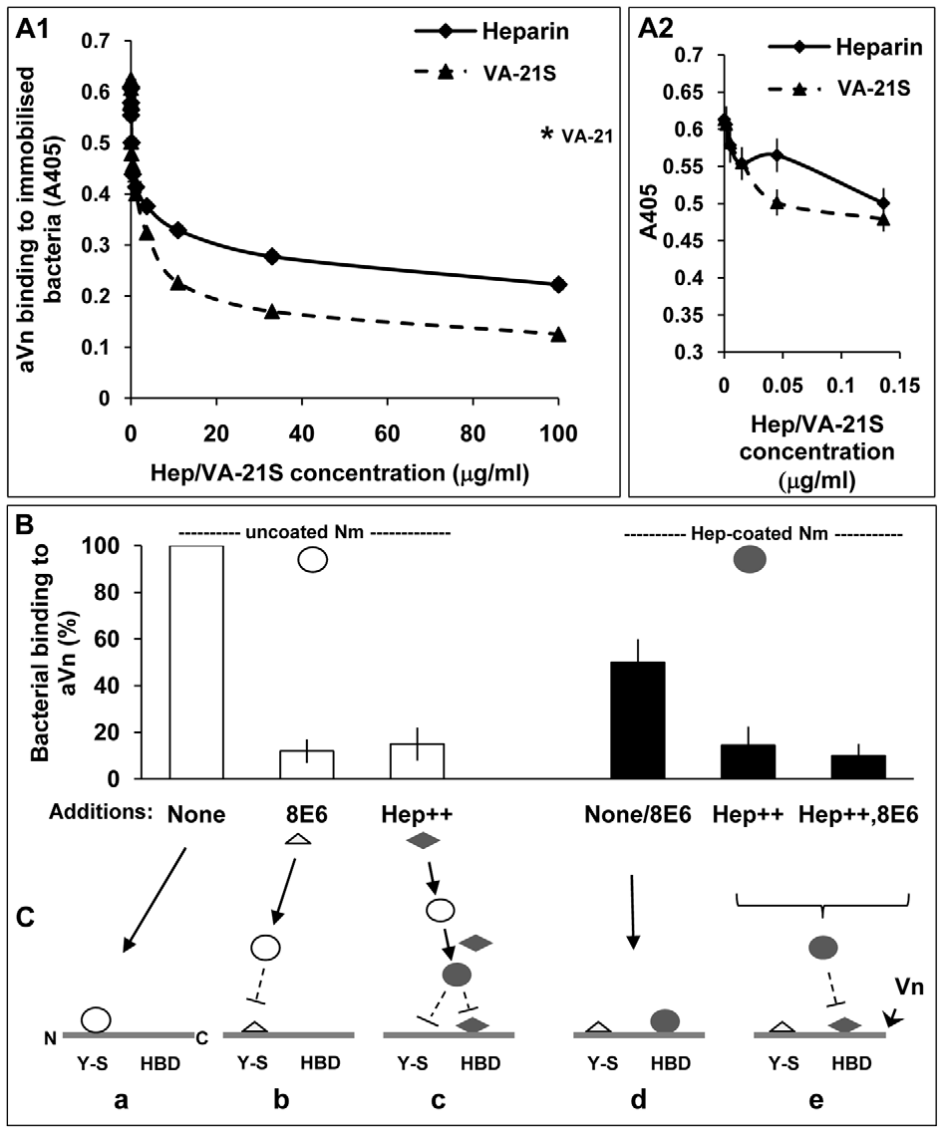
Biphasic effect of heparin on bacterial interactions with human vitronectin. ELISA plates with immobilised acapsulate Opc^+^ Nm were blocked with BSA block (pre-filtered using heparin-sepharose and DEAE-sephacel) and then overlaid with heparin or VA-21S peptide at the concentrations shown for 20 min prior to the addition of aVn (2.5 µg/ml). Vitronectin binding was assessed as described in Methods. In A1, the results observed over the full range of concentrations are shown whereas in A2 data at a narrower range of heparin concentrations are depicted and VA-21S data are included for comparison. The level of binding in the presence of the control peptide VA-21 when used at a concentration of 100 µg/ml is shown by an asterisk in A1. Data shown are representative of two independent experiments performed using bacteria grown either on GC-agarose or HBHI. Identical results were obtained in both cases. (B) ELISA plates with immobilised aVn were overlaid with uncoated or heparin-precoated bacteria in the absence or presence of excess 8E6 or heparin (Hep^++^) or both (Hep^++^,8E6) during the incubation. Bacterial binding was assessed using polyclonal anti-Nm antibody. In each case, percent binding to Vn relative to the highest value observed (i.e. uncoated bacteria in the absence of inhibitors) are shown. (C) The sequence of events consistent with the data in B is represented in a schematic diagram. The horizontal lines represent unfolded Vn molecule (Vn) with its N terminal ‘Y-S’ domain and C-terminally located heparin-binding domain (HBD). These regions may constitute two separate binding sites for Nm on Vn (triangles: mAb 8E6, diamonds: heparin, open circles: uncoated Opc^+^ Nm, filled circles: heparin-coated Opc^+^ Nm). a: uncoated Nm bind directly to the Y-S region; b: the mAb 8E6 also binds to Y-S and blocks binding of Nm to the site; c: excess heparin present in the medium coats bacteria and prevents binding to Y-S and in addition, it binds to the HBD and prevents hep-coated Nm binding to HBD; d: Nm precoated with heparin cannot bind to Y-S but in the absence of excess heparin, can bind to HBD; in this case added mAb 8E6 does not affect the level of Nm adhesion to Vn; e: excess free heparin added to the medium binds to HBD and blocks heparin-coated Nm binding to the site; again the presence of mAb 8E6 binding at the CR of Vn has no effect.

In an attempt to analyse the relative preference for bacterial binding directly to the Y-S of Vn or HBD through heparin, experiments were performed using heparin and VA-21S as inhibiting agents. Inhibition of aVn binding to bacteria was observed at increasing heparin as well as VA-21S concentrations (Figure 8A1), this may suggest a common site on Opc for binding directly to Vn and to heparin.

On a closer examination of the effect of heparin when used at low concentrations in ELISA, a biphasic effect was observed: an initial decrease followed by a small rise in binding which was not sustained as heparin concentration increased and eventually there was an overall decrease in bacterial binding to aVn (Figure 8A2). This biphasic effect of heparin suggested that in the presence of low levels of heparin, two competing interactions with aVn may occur. Some bacterial binding may continue to occur at the tyrosine sulphated site; in addition, binding to HBD via the bound heparin may also come into play. An interesting observation from this study was that the presence of heparin did not produce more binding to aVn than observed in its absence at any concentration. This might suggest that while the presence of limited amount of heparin in an ELISA may alter the site of bacterial binding on aVn, this alternative binding is of a lower affinity than the binding to sulphated region of Vn CR.

#### B306 as a modulator of direct and indirect binding

As implied in the above studies, if Nm Opc contains a single or overlapping binding site/s for both Y-S and heparin, then the indirect Vn-binding might also be expected to be inhibited by the anti-Opc mAb B306, which inhibits the direct binding (Figure 7E). To assess the effect of B306 on heparin-mediated binding, first bacteria were coated with B306 and then with heparin. These bacteria adhered to aVn at the same level as those coated with B306 alone. Similarly, in a reverse coating sequence, bacterial adherence levels were dictated by the first coating of heparin (not illustrated). These observations suggest that B306 also inhibits heparin interactions with Opc.

### Dual binding sites of Opc on activated vitronectin

To finally demonstrate the dual mechanisms of Vn targeting by Opc, we raised and tested the following hypothesis, taking into account that a single site of Opc may bind to the sulphated regions of Vn and heparin. If Opc^+^ Nm were precoated with heparin and the excess removed, bacterial adhesion could only occur via the heparin-binding domain/s of Vn as the Y-S-binding region of Opc would be fully occupied with heparin. In the presence of excess heparin, the HBD domain of Vn should also be saturated. This should prevent all Opc interactions with Vn. However, it has to be noted that an induced-fit mechanism of ligand-targeting might be involved in binding of Opc to its ligands [19]; in this case, some differences may exist in binding of Opc to the CR of Vn and to heparin.

**Figure 9:**
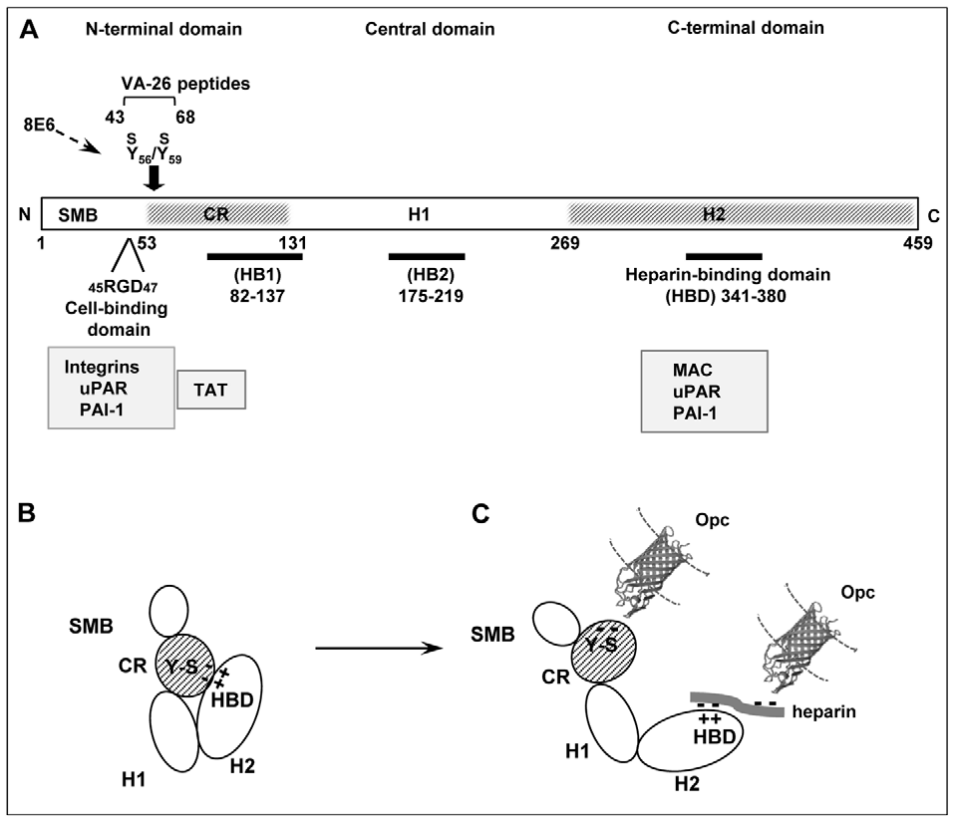
Vitronectin domain structure, conformational states and some physiological and bacterial ligand binding sites. (A) A schematic presentation of linear vitronectin structure showing the positions of the cell-binding motif RGD, sulphated tyrosines (Y_56_S/Y_59_S) and the high affinity heparin-binding domain (HBD), the three regions of particular importance in meningococcal interactions with host receptors. The N-terminal domain contains a region homologous to somatomedin B, (SMB, residues 1–44). The central and the C-terminal domains contain regions homologous to haemopexin (H1 and H2). An extended connecting region (CR) joins the N-terminal and the central domains and contains a stretch of acidic residues (53–61) within which two sulphated tyrosines (Y-S) are located. This site is cryptic in the native vitronectin and comprises the mAb 8E6 binding epitope requiring the presence of sulphated residues [30]. The C-terminal domain residues 341–380 contain the main heparin-binding domain (HBD) and are composed of several highly charged residues [26]. Arginine at positions 351/353 may bind directly to heparin [45]. Two other sites implicated in heparin binding (but with low affinity) are shown as HB1 and HB2. Several pathogens bind to the central domain and via heparin to HBD [46], [64], [65]. Only Nm has been shown in our current study to bind to the Y-S region in the vitronectin connecting region. Binding positions for physiological ligands are shown (grey boxes). Information is partly based on [23], [26], [27] and references there in. (B) An illustration of the folded conformation of the vitronectin molecule which may bring the acidic sulphated tyrosines and the basic HBD in close proximity [23]. (C) Under physiological or *in vitro* denaturing conditions, Vn structure is relaxed exposing fully the HBD binding site and making available the mAb 8E6 epitope in the CR, both with the capacity to bind to *N. meningitidis* Opc protein. The binding at the HBD may additionally be assisted by the multimerisation of Vn, which increases the affinity of multimeric Vn for heparin [27]. The molecular model of Opc was kindly provided by Prof. Jeremy Derrick. The Opc crystal structure has been described by Prince *et al*. [17]; this study also presents a model of the possible mechanism of interaction of the Opc surface exposed loops with heparin-like proteoglycans.

To test this hypothesis, aVn was overlaid with uncoated or heparin-precoated bacteria in the absence or presence of excess 8E6 to block the Y-S site or excess heparin to block the HBD of Vn. The direct binding of uncoated Opc^+^ Nm was inhibited by the addition of 8E6 as well as in the presence of excess heparin. Pre-coating Opc^+^ bacteria with heparin before measuring their binding to activated Vn shows lower binding (by ∼50%) than uncoated bacteria (4^th^ bar, Figure 8B) suggesting a lower affinity of binding through this route. In addition, unlike uncoated bacteria, the presence of 8E6 does not affect the level of binding of heparin-precoated bacteria (also represented by the 4^th^ bar, Figure 8B). As the mAb 8E6 binds to the Y-S region, it may not be expected to interfere with Nm binding at the distally located HBD domain in the activated form of Vn. Excess heparin (with or without 8E6) itself by binding to HBD, competes with heparin-coated Nm and almost completely inhibited bacterial binding to Vn in accordance with the above hypothesis. This is consistent with the binding of coated and uncoated bacteria at distinct regions of Vn. In addition, the direct binding to sulphotyrosine region of Vn has already been established above (Figure 6). A proposed sequence of events consistent with the data is illustrated in Figure 8C. Figure 9 summarises some of the Vn properties and illustrates the binding sites for Opc^+^ Nm on activated human vitronectin.

## Discussion

For a number of human pathogens, extracellular matrix and serum proteins serve as mediators of anchorage to host cell surfaces, a process that often leads to cellular invasion [1], [2], [4], [48], [49]. Fibronectin and vitronectin have been shown repeatedly to serve as such molecular bridges. However, their binding by microbes may itself occur indirectly via heparin, as Fn and Vn are both heparin-binding proteins and many bacteria can bind to heparin-like negatively charged polymers [46], [50], [51].

Only a limited number of studies have been carried out on Nm Opc and its requirements for binding to Fn/Vn and subsequent attachment to human cells. Some pertinent observations from these studies are outlined below. It appears that Nm Opc and a related but distinct class of neisserial adhesins, the Opa proteins, may directly bind to Fn, Vn was not investigated in this study [52]. Eberhard *et al.* [52] also observed the direct binding of several Opa^+^ Opc^+^ capsulate strains of Nm to the central cellular binding domain of Fn. Further, they demonstrated similar binding of Nm isolates to immobilised cFn and pFn but much reduced interactions were observed when pFn was provided in solution. As cFn and pFn have structural differences, their interactions with bacteria may differ. In addition, immobilisation of pFn may also lead to altered conformation of the molecules which may affect observed binding levels.

On the other hand, other studies have illustrated a possible requirement for sulphated bridging ligands for Nm Opc/Vn interactions [46]. These studies used dextran sulphate as a substitute for heparin which increased Opc binding to Vn significantly over that observed in the absence of any bridging molecules. *N. gonorrhoeae* Opa proteins also required heparin or dextran sulphate; notably, dextran sulphate supported stronger interactions with Fn and Vn than heparin [46].

To define the role of Opc, in our previous studies, defined acapsulate isolates of Nm strain C751 and MC58 either expressing or lacking the expression of Opc, direct Nm Opc-mediated binding to Vn was shown, which resulted in increased adhesion and invasion of HUVECs [1], [2]. Our preliminary observations also suggested that Opc may bind directly to purified Fn and its binding to purified cFn was somewhat better than to the pFn preparation employed [1]. More recent studies of Unkmeir *et al*. (2002) that used Opa and pili-expressing capsulate phenotypes of Nm, did not observe a role for Vn in mediating HBMEC adhesion or invasion, rather an inhibition of basal cellular invasion was reported in the presence of purified Vn. Further, when using purified fibronectins, cFn appeared to be the more efficient form in increasing bacterial invasion of HBMECs. Notably, there are several differences in the choice of assay designs, cell lines and bacterial phenotypes in the above investigations that may account for the differences between the above observations. However, our unpublished observations have shown that significant variations can also occur in purified preparations of serum proteins from distinct sources and may account for some of the reported observations.

### Sialylation and cellular interactions

It is well documented that in non-piliated Nm, the capsule and sialylated LPS dampen the interactions of subcapsular adhesins with their cell-expressed receptors [2], [3], [20]. However, in this setting, additional expression of pili can overcome such dampening effects to bring the outer membrane adhesins and host cell receptors in close apposition [6], [7], [8].

In order to assess the potential of Opc in capsulate as well as acapsulate bacteria to bind to Fn or Vn conformers present in human serum, in the current study, we employed two distinct meningococcal strain isolates: those of Nm strain C751 with no surface expression of capsule and those of strain MC58 with capsule and intrinsically sialylated LPS. The latter also expressed pili, Opa and Opc, representing a phenotype of relevance in *in vivo* settings. The data show that the Nm Opc in such acapsulate and capsulate bacteria can bind directly to human serum factors and mediate endothelial cell adhesion and invasion. Our unpublished studies also show that, in capsulate Nm, interactions of subcapsular adhesins with the soluble serum effectors (e.g. Vn) are not dependent on pilus expression; whereas their further interactions with cellular receptors are facilitated by the presence of pili. Such a role of pili in initiating the initial cellular engagement and leading to facilitation/potentiation of the interactions of Nm with human cells via integral outer membrane proteins has been established in our previous studies on epithelial cells [6], [7], [8].

Overall, from the current studies, the levels of serum Fn bound to Opc^+^ Nm appear to be lower than of serum Vn and consequently serum-dependent HBMEC adhesion and invasion are significantly inhibited with 8E6, an aVn-specific mAb. In addition, none of the purified Fn preparations supported adhesion or invasion to the extent observed either with serum or certain purified Vn preparations (i.e. aVn). These data confirm that Opc can bind to human fibronectin but also suggest that Vn may be the preferred target, as observed previously [1].

### Opc^+^ Nm interactions with vitronectin

Vitronectin occurs in two distinct conformations, a native conformation with a cryptic heparin binding site and an unfolded, active conformation with fully exposed binding sites for heparin/heparan sulphate and the mAb 8E6 (see Figure 9 and associated citations). In our earlier studies it had been noted that some, but not all, preparations of Vn, supported efficient direct binding of Opc-expressing Nm to Vn (unpublished observations). As Opc is a heparin-binding protein and as it has been implied that Opc may require sulphated bridging molecules to bind to Vn [46], detailed investigations were undertaken to assess the direct as well as the indirect binding requirements for Opc^+^ Nm and the potential role of Vn in supporting adhesion and invasion of endothelial cells of human brain origin.

### The role of vitronectin sulphation in Nm interactions

Opc may bind to Vn and Fn via a heparin bridge [20], [46]. However, Nm Opc clearly also interacts with Vn directly, as this occurs in the absence of added heparin. Notably, growth conditions did not result in prior acquisition of sulphated contaminants (as occurs with *Neisseria gonorrhoeae* [47]), that could account for this observation, which could be specifically inhibited by anti-Vn mAb 8E6 and by heparin. The mAb 8E6 has been shown unequivocally to bind to the CR of Vn and require the sulphation of the tyrosine residues Y_56_ and Y_59_ in the Vn connecting region for efficient interactions with Vn [22], [30], [53]. However, 8E6 may inhibit bacterial binding via steric hindrance as in the case of its inhibition of the urokinase-like plasminogen activator receptor (uPAR) which binds to a region in the nearby somatomedin B domain [30]. Further evidence for the involvement of the 8E6 epitope as a major binding site for Nm Opc came from the use of acid-desulphated Vn which no longer supports either Opc^+^ bacterial binding or mAb 8E6 binding presumably due to the loss of acid-labile O-linked sulphated residues [30]. Acid desulphation under mild conditions does not destroy the protein antigenicity in general as shown in the current study (Figure 5). Studies from other groups also report that acid desulphation does not alter uPAR or PAI-1 binding to Vn [29]. It is also worth noting that any phosphorylated amino acids present (such as threonine 50 and 57) may not be affected by the acid-treatment employed as O-linked phosphates are highly resistant to acid hydrolysis [42], [43] (as also demonstrated in Figure S1).

In addition, in our studies, sulphated peptide spanning the Vn residues 48–68 (VA-21S) or the biotinylated VA-26S (spanning the residues 43-68) significantly inhibited the binding of Opc^+^ Nm to aVn. The equivalent non-sulphated peptides (VA-21 or VA-26) were only weakly inhibitory. Further, we also analysed the effect of a synthetic peptide of Vn modified at Thr_50_ and Thr_57_ with phosphate groups (VA-26P), as Vn can be modified at these residues by the action of CK2 [39], [40], [41]. This peptide did not inhibit bacterial binding to aVn even at high concentrations. The VA-21/S peptides could not be immobilised effectively on to solid surfaces; therefore the biotinylated VA-26/S/P peptides tethered to extravidin immobilised on ELISA plates were used to assess the direct binding of Nm. In this system also, Opc^+^ Nm binding to VA-26S but not to VA-26 or to VA-26P could be seen. The data show clearly that Opc^+^ Nm can target residues within the connecting region of vitronectin and this is facilitated by the sulphated tyrosines of the connecting region.

### The minimal Vn features required for cellular invasion by Opc-expressing meningococci

The RGD motif in the 26-mer peptides also enabled immobilisation of the peptides on the endothelial integrins and examination of the adhesion and invasion-supporting roles of the 26-mer VA-26/S/P peptides. From these studies it is clear that sulphated tyrosines and the RGD motif as in VA-26S peptide are required for mediating Opc-dependent cellular adhesion and invasion of both capsulate and acapsulate meningococci.

### Tyrosine sulphation of proteins

Tyrosine O-sulphation is a common post-translational modification of secretory and membrane-bound proteins which takes place in trans-Golgi network and is catalysed by membrane-anchored tyrosylprotein sulphotransferases. The classes of proteins that contain sulphotyrosines include G protein–coupled receptors, adhesion molecules, coagulation factors, hormones and extracellular matrix proteins [31], [54]. In vitronectin, the residues Y_56_ and Y_59_ have been unambiguously determined as the tyrosine sulphation sites [29], [30], [31]. It has been proposed that the sulphated tyrosine residues of Vn have a role in its conformational stability as the highly acidic tyrosine sulphated region binds intramolecularly to the highly basic heparin-binding region at the C-terminal end of Vn thereby locking vitronectin in an inactive conformation (as depicted in Figure 9) [29], [31].

There is increasing evidence that sulphotyrosines partake in protein-protein interactions in the extracellular space and the modification has been shown in many cases to be required for efficient ligand-receptor interactions. Its importance has been reported particularly for chemokine receptor functions [55]. Interestingly, HIV enters macrophages and T cells via interacting with sulphated residues of the chemokine receptor CCR5 [56]. However, to our knowledge, such an involvement of sulphated tyrosines in bacterial targeting of host receptors and pathogenesis has not been previously reported.

### Opc binding to Vn via heparin

As sulphated residues are involved in mediating both the direct binding to the CR and in indirect binding to HBD, it is very likely that the same region of Opc is involved in these two interactions. If a common site binds to the sulphates of these diverse receptors, it may be envisaged that, at certain low heparin concentrations, bacterial binding to the HBD could occur via a heparin bridge and, a level of bacterial binding could also occur directly to the sulphated tyrosines via Opc sites unoccupied by heparin. On the other hand, in the presence of excess heparin, all Y-S binding sites on Opc may be occupied and in addition, the HBD on Vn may also be blocked by heparin. Thus, Opc-mediated interactions may be totally inhibited. This was indeed the case (data in Figure 8). In addition, in the absence of any free heparin, heparin precoated bacteria (which can only bind to Vn via the HBD) bind less effectively to Vn than uncoated bacteria suggesting that binding to HBD via heparin is of a lower affinity than direct binding to the Y-S site in the Vn connecting region.

As iterated above, in previous studies to demonstrate the requirement for sulphated ligands in indirect Opc/Vn interactions [46], low levels of Opc^+^ Nm binding to Vn occurred in the absence of added ligands. However, in the presence of dextran sulphate relatively higher binding to Vn was observed. Notably, in these studies, heparin was less efficient than dextran sulphate for the indirect binding of Opa-expressing *N. gonorrhoeae* to Vn and Fn. Thus, affinities of such different sulphated ligands for Opc (and Opa) may vary considerably and in turn affect the efficacy of vitronectin targeting. As our current studies have used heparin solely, we can only draw conclusions about the relative affinities for heparin-dependent and independent interactions of Opc with Vn.

It is pertinent to note also that Opc^+^ Nm binding to native Vn did not change in the presence of added heparin (data not illustrated), and the binding levels of Opc^+^ and Opc^−^ phenotypes were similar to those shown in Figure 4 for direct Opc^+^ Nm interactions with native Vn. This suggests that the activated, unfolded vitronectin is the only target for effective direct or indirect binding of Nm.

### Species specificity

Vitronectin is evolutionarily highly conserved [23]. Examination of the sequences within the CR of human, bovine and murine vitronectins revealed that Y_56_ is conserved in all three proteins but Y_59_ is not present in bovine Vn. However, we observed experimentally that the mAb 8E6 binding requirements were only present in human Vn. Nonetheless, Opc^+^ Nm binding to both bovine and murine vitronectins was found to be significantly higher compared with Opc^−^ Nm. As acid hydrolysis destroyed these interactions, Y_56_S residue and other tyrosines located at Y_59_/Y_61_ may significantly contribute to Nm binding to bovine and murine vitronectins also. Tyrosine sulphate modification of proteins is not uncommon among eukaryotic proteins and it has been observed that sulphated tyrosines are often grouped together in defined clusters and are frequently flanked by acidic residues [54]. The environments in the bovine and murine vitronectins are thus favourable for sulphation and for bacterial binding but apparently not for 8E6 binding, which clearly has a more strict epitope requirement; accordingly, the mAb has a very limited number of targets especially in human serum [53]. We also observed that, the anti-Opc mAb B306 against the Opc loop 2 inhibited Opc^+^ Nm binding to all three vitronectins suggesting that the adhesin binds to the three proteins using largely similar mechanisms. Finally, VA-21S (with human Vn sequence) also significantly inhibited Nm Opc interactions with the animal-derived vitronectins, also suggesting similar mechanisms of binding to the three vitronectins. Further site-directed mutagenesis of both Opc and of various animal/human vitronectins are required to finally confirm the observations.

### *In vivo* implications

Vitronectin is one of the more abundant plasma proteins circulating at ∼200–400 µg/ml in humans and makes up 0.2–0.5% of total plasma proteins [23], [26]. Only a small proportion (∼2%) has been estimated to be in its activated state in plasma but after coagulation, in serum this may increase to ∼7% [33]. However, conformational changes in the protein occur *in vivo* as it becomes activated by the interactions of circulating ligands such as TAT and MAC complexes which increase during pathogenic conditions. Among other functions, Vn serves as scavenging receptor for the end products of the haemostasis and complement systems and complexed Vn is rapidly cleared from circulation [23], [57]. Increased native Vn consumption due to continuous activation of Vn during disseminated Nm infections may indeed occur [28]. The availability in several such complexes for the mAb 8E6 binding site [28], [36] suggests that Nm Opc may also be able to target any activated Vn sequestered into the complexes, which is under investigation. The frequent conversion of native Vn to activated Vn ensures a constant supply for bacterial binding which could lead to increased cellular interactions at the brain and vascular endothelial interfaces and an increased potential to traverse the barriers as observed *in vitro*. This study has demonstrated that capsulate meningococci can interact with HBMECs via Opc/Vn bridge; the studies of Unkmeir *et al*. [4] have shown this to be the case via Fn. Whether such interactions occur *in vivo* remains to be shown. As murine Vn may support Opc binding, suitable animal models could be used to assess this phenomenon. However, it is noteworthy that the efficacy of subcapsular adhesins in mediating cellular interactions of capsulate phenotypes may only be effectively observed when the bacteria are also piliated (as discussed above). Accordingly, with respect to pilus receptor expression, humanised animal models may be required in order to assess the *in vivo* significance of the reported findings. Finally, as reported for other pathogens, in addition to increased cellular interactions, vitronectin binding could also enable bacteria to become more serum resistant (N. J. Griffiths and M. Virji, manuscript in preparation; [58]). Thus, the targeting of activated vitronectin may impart to Opc-expressing meningococci the properties of efficient tissue penetration and resistance to host innate and adaptive immunity required for effective dissemination.

## Materials and Methods

### Bacteria and growth conditions

*N. meningitidis* phenotypes with or without the expression of Opc (Opc^+^, Opc^−^) were derived from the serogroup A strain C751 and have been characterised and described previously [3], [10]. These isolates are acapsulate and do not express the other major adhesins Opa or pili. Their outer membrane profiles by SDS-PAGE appear identical other than in the expression of Opc protein. They express pilin monomers which are incorporated in the outer membranes but these are not assembled into pili by EM and are not functionally effective [3], [7]. Their reactivity with a number of anti-LPS mAbs is also identical, they possess lactoneotetraose in LPS which can be only extrinsically sialylated in serogroup A Nm. As blood isolates are usually capsulate and express pili and the opacity adhesins Opa and Opc, to investigate the role of Opc in adhesion and invasion of endothelial cells by such capsulate bacteria, Opc^+^ and Opc^−^ isolates of serogroup B MC58 strain were used which were otherwise replete with capsule, pili and Opa expression. These isolates express L3 LPS immunotype which can be intrinsically sialylated [6].

All meningococci were normally grown on brain-heart infusion agar supplemented with heated horse blood (HBHI) [35]. This medium contains some sialic acids from the blood supplement and so the LPS of the serogroup A strain C751 can become extrinsically sialylated. In some experiments where indicated, a defined GC-agarose medium (utilising ultrapure agarose as the gelling agent [59]) was used for the growth of this strain. In this case, no serum supplement is present, thus there is no source of sialic acid for incorporation into LPS. Notably, no qualitative or significant quantitative differences in Vn targeting were observed when using C751 isolates grown on these two media (Figure S4).

### Cell lines

Human brain microvascular endothelial cell line (HBMEC) was kindly provided by Dr K. S. Kim, John Hopkins University, USA [60]. Cells were grown in RPMI-1640 medium supplemented with 15% (v/v) heat-inactivated foetal calf serum (FCS) (Cambrex), 2 mM glutamine, 1 mM sodium pyruvate, 100 U ml^−1^ penicillin-streptomycin, 1% (v/v) MEM non-essential amino acids solution and 1% MEM vitamins solution at 37°C in 5% CO_2_.

### Antibodies

Anti-Vn mAb clone 8E6 (Millipore) was used to detect and block the interactions of activated Vn at 1–5 µg/ml and 10 µg/ml respectively. The monoclonal anti-Vn antibody VIT-2 and polyclonal anti-Fn antibodies were obtained from Sigma and polyclonal anti-Vn antibody was from Millipore (AB19014). Anti phosphothreonine (Sigma) (anti-Thr-P) was used at a range of dilutions as indicated to detect phosphorylation of threonine residues in the synthetic peptide VA-26P. Anti-Opc mAbs B306 and 154,D-11 were kindly provided by Prof. M. Achtman and Dr. J. Kolberg respectively [61], [62]. Where not stated, the antibodies were used at predetermined optimum concentrations (usually, 1–10 µg/ml).

### Purified proteins and synthetic peptides

Native Vn was purchased from Molecular Innovations. Fibronectin and activated Vn from normal human serum (purified using heparin-sepharose chromatography) were obtained from Sigma. Synthetic peptides corresponding to the Vn residues 48–68 (VA-21) and the corresponding sulphated peptide (VA-21S) were used in competition experiments. As these could not be immobilised efficiently to solid surfaces, biotinylated peptides were also used. These peptides spanned the Vn residues 43–68 (VA-26) and at positions 45–47 they contained the RGD cell binding sequence as in Vn. The unmodified VA-21, VA-26 and the phosphorylated VA-26P were obtained from GL Biochem (Shanghai, China). The sulphated peptides VA-21S and VA-26S were synthesised by Cambridge Research Biochemicals (Cambridge, UK).

### Bacterial binding to serum proteins

#### Heat-treatment of NHS

Decomplemented serum was used in the reported experiments to suppress any bactericidal effects. However, although such heat treatment is known to convert native vitronectin to an unfolded state, the decomplemented serum still contained a substantial level of native Vn as assessed by heparin sepharose fractionation in which aVn can be differentially removed as nVn does not bind to heparin efficiently [23].

Relative binding of Opc to serum Vn and Fn was assessed by incubating 1×10^10^ live Opc^+^ or Opc^−^ Nm with 10% decomplemented NHS. Bacteria were then harvested, washed three times prior to the analysis for bound serum proteins. Bacterial lysates were subjected to SDS-PAGE and western blotted, and the presence of the proteins detected using anti-Vn or anti-Fn antibodies.

For assessment of the preference for binding to activated Vn, native Vn or Fn, their depletion from serum was analysed by sequential adsorption of a serum sample with three freshly prepared live bacterial suspensions. Incubations were carried out for an hour and an aliquot of the serum sample was retained. The rest of the sample was subjected to further adsorption with fresh bacterial suspensions. The adsorbed serum aliquots were analysed for the levels of total and activated Vn as well as Fn by immuno-dot blot assays as described below.

### Measurement of bacterial adhesion and invasion

Total cell association (adhesion) and invasion were measured by viable count assays as described previously [35]. Briefly, for cell association, bacterial suspensions were incubated with cell monolayers at an MOI of 300∶1 in medium 199 supplemented as required with pre-determined optimum concentrations of serum or purified proteins and peptides for an infection period of 3 h. After this, target cells were lysed in a 1% saponin solution, diluted and plated. Levels of bacterial invasion were determined by gentamicin protection assays and verified by the use of cytochalasin D [10], [35]. Optimum concentrations of supplements required to support adhesion and invasion were found to be 10% for decomplemented NHS and 10 µg/ml of the purified serum components used. To unfold Vn preparations, in some experiments, samples of purified Vn preparations (human nVn, bovine and mouse Vn samples) were treated by heating at 56°C for 30 min. The details of experiments to test the effects of Vn peptides or antibodies on bacterial interactions with HBMECs are described in the text.

### Immunofluorescence assays

In some experiments, bacterial adhesion levels were visualised by microscopy after fluorescent labelling of bound bacteria as described previously [63]. Briefly, cell monolayers with adherent bacteria were fixed with methanol and non-specific binding sites blocked with 3% BSA in phosphate buffered saline containing 0.05% Tween-20 (PBST). Bacteria were detected by overlaying with a rabbit polyclonal antiserum raised against Nm and secondary antibodies conjugated to rhodamine.

### Immuno-dot blot assays

Activated Vn, native Vn, or heat-treated nVn were dotted onto nitrocellulose at a starting concentration of 375 ng/dot followed by serial 2-fold dilutions, and non-specific binding sites blocked with 3% BSA-PBST. Nitrocellulose strips were then overlaid with anti-Vn antibodies and their binding was detected using alkaline phosphatase (AP)-conjugated secondary antibodies. In some experiments bacterial binding to immobilised Vn was assessed using Opc^+^ Nm (1×10^10^ bacteria/ml) which were allowed to bind to immobilised Vn for 2 h. Bacterial binding was detected using polyclonal antiserum against Nm and AP-conjugated secondary antibodies. Relative levels of antibody binding were ascertained by densitometry using Scion Image software (Scion Corporation, Maryland USA).

### Acid hydrolysis of vitronectin and peptides

Vitronectin samples and biotinylated peptides were treated with 1 M HCl to remove O-linked sulphate groups from the tyrosine residues [30]. In addition, the effect of such acid treatment on phosphorylated peptides was also monitored. Briefly, 2.5 µg of the appropriate Vn stock or 5 µg of VA-26S/VA-26P biotinylated peptides were incubated for up to 20 min at 80°C in 1 M HCl before neutralising with 1 M Tris. The level of sulphation of the human aVn (or sulphated peptides) was then determined by immuno-dot blot using the mAb 8E6. Phosphorylation status of VA-26P after acid treatment was monitored by the use of anti-Thr-P antibody.

### Enzyme linked immunosorbant assay

ELISA plates (96 well, Dynex) were incubated overnight with purified Vn in PBS or in carbonate buffer pH 9.5. For direct binding studies, Opc^+^ Nm were added at 10^8^ bacteria/well in PBS. The role of heparin was investigated by pre-coating bacteria with 50 µg/ml heparin (Sigma) for 30 min, followed by washing to remove excess heparin prior to the addition to wells in the presence or absence of an additional 50 µg/ml heparin, which was present throughout the initial binding period. In all cases, bacterial binding was detected by the addition of a rabbit polyclonal antiserum raised against Nm followed by AP-conjugated secondary antibody. All antibodies were incubated for 1 h at room temperature in 1% BSA-PBS. Plates were developed using SigmaFast p-Nitrophenyl phosphate substrate and absorbance was measured at 405 nm (A405). Alternative ELISA procedures for detecting the binding of Vn to immobilized bacteria were also performed and the Vn binding detected with polyclonal anti-Vn antibody and developed as above. Where heated native Vn was used for overlay, samples were heated for 30 min at 56°C prior to addition to wells. For biotinylated peptide ELISA, plates (as above) were incubated overnight with 10 µg/ml extravidin (Sigma). Biotinylated peptides were then immobilized at concentrations described in the text, for 1 h. Unbound peptides were removed by washing. In some tests, peptide loading was assessed using the mAb 8E6 or anti-Thr-P antibody as described above or using competitive ELISA as described in Figure S2. Peptide-containing ELISA plates were used to assess the direct bacterial binding at a variety of peptide and bacterial concentrations. Bound bacteria were detected by appropriate antibodies as above.

### Statistical considerations and data presentation

In general, one typical of several independent experiments is shown. At least triplicate determinations were included within each experiment and mean values with standard errors have been shown.

## Supporting Information

Figure S1 Acid hydrolysis of Vn peptides removes sulphated residues specifically. To test the effect of acid treatment on the stability of sulphate modification vs. phosphate modifications of the biotinylated vitronectin peptides, acid treated and untreated peptides were immobilised (5 µg/ml each) on ELISA plates pre-loaded with 10 µg/ml extravidin and the levels of sulphation and phosphorylation assessed using antibodies 8E6 and anti-Thr-P respectively. The data demonstrate the specific removal of sulphates with the treatment, as 8E6 binding is diminished but not anti-Thr-P binding.
(0.14 MB TIF)

Figure S2 Immobilisation of biotinylated peptides on extravidin ELISA plates. (A) Optimum concentration of extravidin for coating ELISA plates was pre-determined and was found to be 10 µg/ml. The levels of biotinylated peptides immobilised on extravidin plates were assessed using different concentrations of VA-26S and VA-26P biotinylated peptides. The concentration dependent binding of VA-26S is illustrated. In addition, the maximum binding of VA-26P is shown which was achieved at ∼5 µg/ml peptide. (B) As no antibodies to detect unmodified biotinylated VA-26 were available, to assess its binding to extravidin plates, competition ELISA was performed using biotinylated peptides as follows: VA26 alone, VA-26S alone or VA-26 followed by VA-26S. Binding of the sulphated peptide was detected using the mAb 8E6. The data show the binding of the sulphated peptide is inhibited by prior coating the plates with the unmodified peptide, which appears to bind as efficiently as the VA-26S peptide to extravidin coated plates.
(0.18 MB TIF)

Figure S3 Opc region/s involved in the direct and indirect targeting of human vitronectin. Two mAbs against Opc were used to assess their inhibitory effects at different concentrations using activated Vn (aVn). A dose-dependent inhibition of binding of aVn to immobilised bacteria in the presence of the anti-Opc mAb B306 against loop 2 was observed but the mAb 154,D-11 against loops 4/5 had no significant effect even at 50 µg/ml suggesting that the Opc binding site for Vn resides close to the antibody B306 binding site on the adhesin, or that B306 restricts the induced fit interaction of Opc with its ligand. Further studies were performed on bovine and murine vitronectins using 30 µg/ml of the antibodies and the data are shown in Figure 7E.
(0.11 MB TIF)

Figure S4 The effect of different bacterial culture conditions on C751 Opc^+^ Nm interactions with vitronectin. Untreated bacteria cultured on HBHI or GC-agarose media were immobilised on to ELISA plates (10^8^ per well). The plates were blocked with heparin-sepharose and DEAE-sephacel-filtered BSA block, and the bacteria were then overlaid with 50 µg/ml of the peptides VA-21S and VA-21 or heparin (used at 33 µg/ml) for 20 min, followed by aVn (added at 2.5 µg/ml) for 1 h. Vn binding was assessed using polyclonal anti-Vn antibody and alkaline phosphatase-conjugated anti-rabbit secondary antibody. In each case, similar results were obtained whether bacteria were grown on HBHI or on GC-agarose indicating that no interfering substances were acquired by C751 isolates during growth on HBHI used in the majority of the experiments.
(0.12 MB TIF)
