# Supplementary figures and images for: Neisseria meningitidis Opc Invasin Binds to the Sulphated Tyrosines of Activated Vitronectin to Attach to and Invade Human Brain Endothelial Cells
Source: PLoS Pathog. 2010 May 20;6(5):e1000911. doi: 10.1371/journal.ppat.1000911 (PMC2873925; doi:10.1371/journal.ppat.1000911)

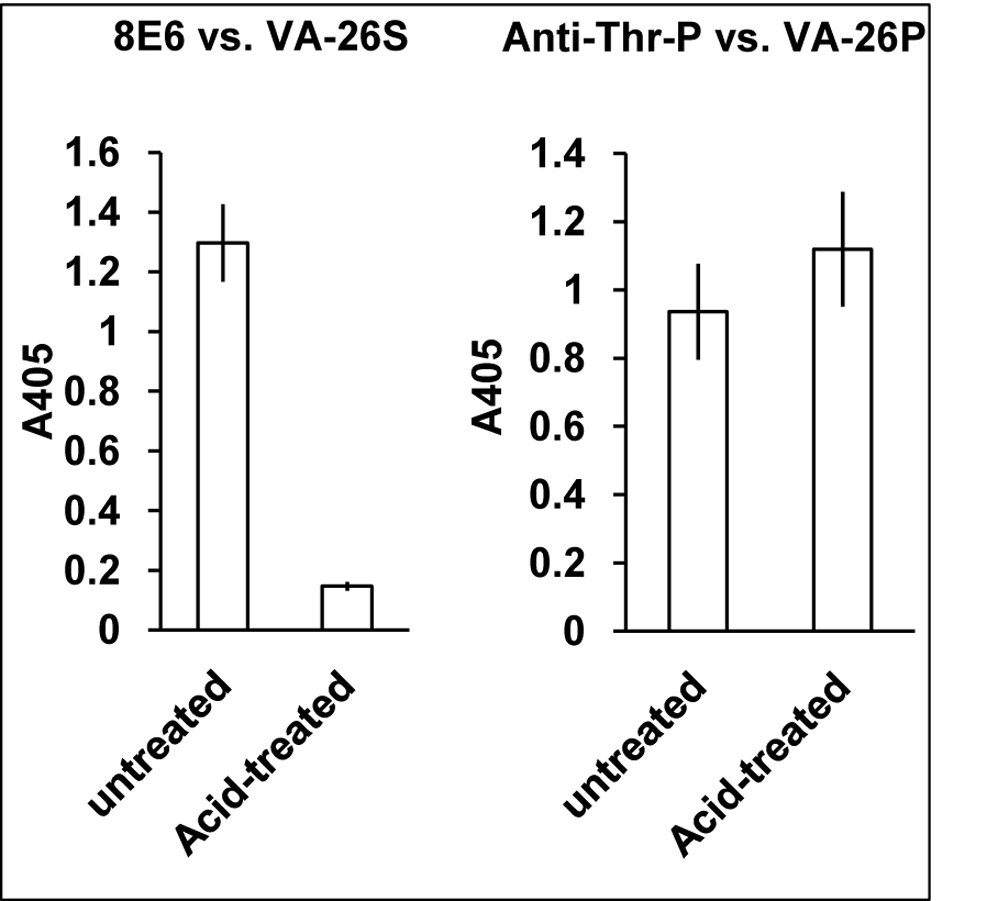

Supplement: Figure S1 — Acid hydrolysis of Vn peptides removes sulphated residues specifically. To test the effect of acid treatment on the stability of sulphate modification vs. phosphate modifications of the biotinylated vitronectin peptides, acid treated and untreated peptides were immobilised (5 µg/ml each) on ELISA plates pre-loaded with 10 µg/ml extravidin and the levels of sulphation and phosphorylation assessed using antibodies 8E6 and anti-Thr-P respectively. The data demonstrate the specific removal of sulphates with the treatment, as 8E6 binding is diminished but not anti-Thr-P binding. (0.14 MB TIF) [file ppat.1000911.s001.tif]

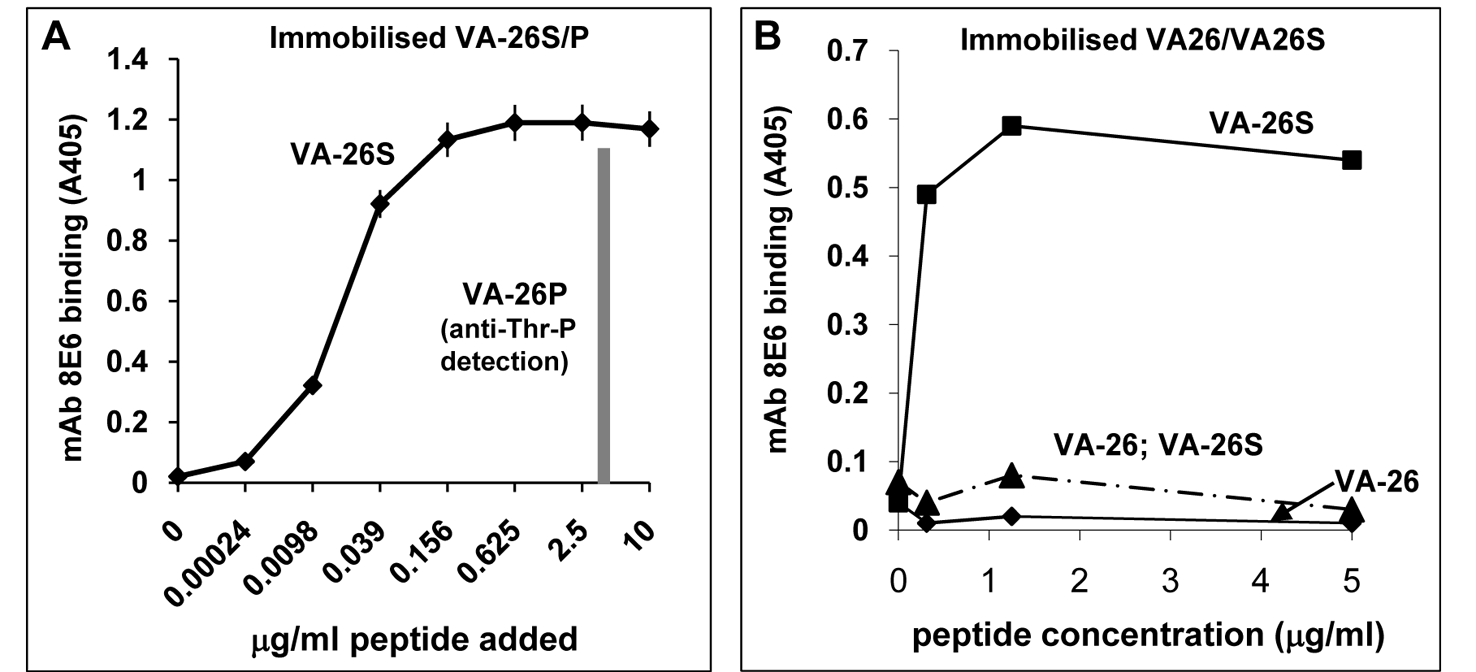

Supplement: Figure S2 — Immobilisation of biotinylated peptides on extravidin ELISA plates. (A) Optimum concentration of extravidin for coating ELISA plates was pre-determined and was found to be 10 µg/ml. The levels of biotinylated peptides immobilised on extravidin plates were assessed using different concentrations of VA-26S and VA-26P biotinylated peptides. The concentration dependent binding of VA-26S is illustrated. In addition, the maximum binding of VA-26P is shown which was achieved at ∼5 µg/ml peptide. (B) As no antibodies to detect unmodified biotinylated VA-26 were available, to assess its binding to extravidin plates, competition ELISA was performed using biotinylated peptides as follows: VA26 alone, VA-26S alone or VA-26 followed by VA-26S. Binding of the sulphated peptide was detected using the mAb 8E6. The data show the binding of the sulphated peptide is inhibited by prior coating the plates with the unmodified peptide, which appears to bind as efficiently as the VA-26S peptide to extravidin coated plates. (0.18 MB TIF) [file ppat.1000911.s002.tif]

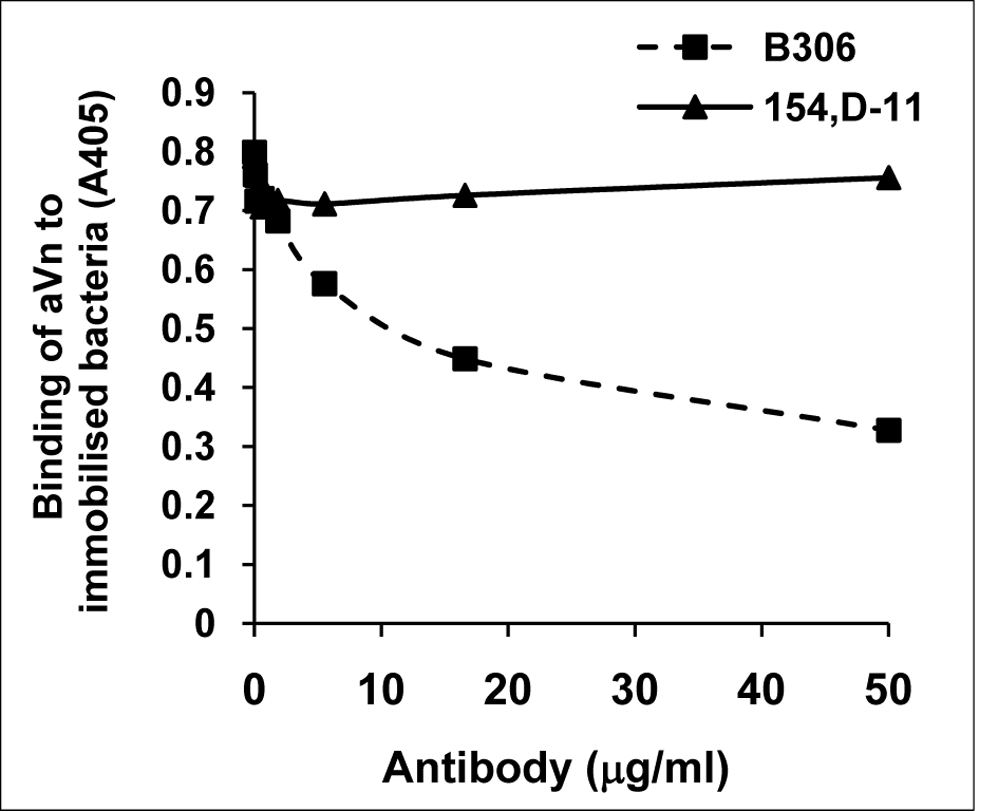

Supplement: Figure S3 — Opc region/s involved in the direct and indirect targeting of human vitronectin. Two mAbs against Opc were used to assess their inhibitory effects at different concentrations using activated Vn (aVn). A dose-dependent inhibition of binding of aVn to immobilised bacteria in the presence of the anti-Opc mAb B306 against loop 2 was observed but the mAb 154,D-11 against loops 4/5 had no significant effect even at 50 µg/ml suggesting that the Opc binding site for Vn resides close to the antibody B306 binding site on the adhesin, or that B306 restricts the induced fit interaction of Opc with its ligand. Further studies were performed on bovine and murine vitronectins using 30 µg/ml of the antibodies and the data are shown in Figure 7E. (0.11 MB TIF) [file ppat.1000911.s003.tif]

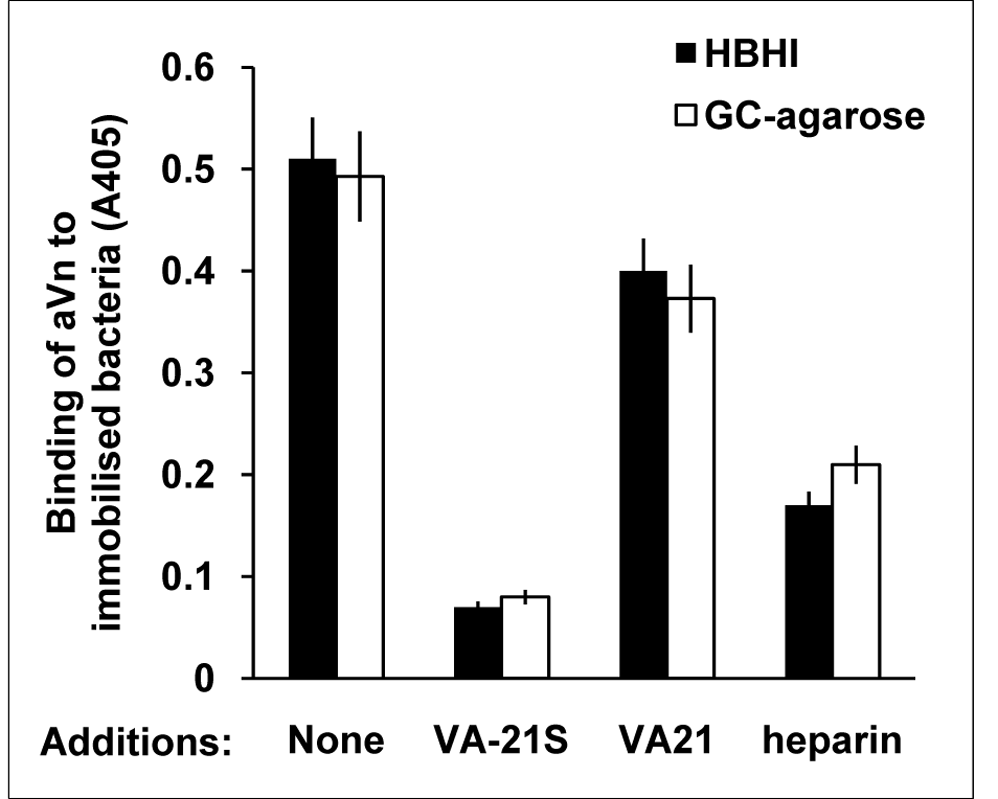

Supplement: Figure S4 — The effect of different bacterial culture conditions on C751 Opc+ Nm interactions with vitronectin. Untreated bacteria cultured on HBHI or GC-agarose media were immobilised on to ELISA plates (108 per well). The plates were blocked with heparin-sepharose and DEAE-sephacel-filtered BSA block, and the bacteria were then overlaid with 50 µg/ml of the peptides VA-21S and VA-21 or heparin (used at 33 µg/ml) for 20 min, followed by aVn (added at 2.5 µg/ml) for 1 h. Vn binding was assessed using polyclonal anti-Vn antibody and alkaline phosphatase-conjugated anti-rabbit secondary antibody. In each case, similar results were obtained whether bacteria were grown on HBHI or on GC-agarose indicating that no interfering substances were acquired by C751 isolates during growth on HBHI used in the majority of the experiments. (0.12 MB TIF) [file ppat.1000911.s004.tif]
